# An endocytic-secretory cycle participates in *Toxoplasma gondii* in motility

**DOI:** 10.1371/journal.pbio.3000060

**Published:** 2019-06-24

**Authors:** Simon Gras, Elena Jimenez-Ruiz, Christen M. Klinger, Katja Schneider, Andreas Klingl, Leandro Lemgruber, Markus Meissner

**Affiliations:** 1 Lehrstuhl für experimentelle Parasitologie, Ludwig-Maximilians-Universität, LMU, Tierärztliche Fakultät, München, Germany; 2 Wellcome Centre for Integrative Parasitology, Institute of Infection, Immunity & Inflammation, Glasgow Biomedical Research Centre, University of Glasgow, Glasgow, United Kingdom; 3 Department of Cell Biology, University of Alberta, Edmonton, Canada; 4 Pflanzliche Entwicklungsbiologie, Biozentrum der Ludwig-Maximilians-Universität, Planegg-Martinsried, Germany; HHMI, Massachusetts Institute of Technology, UNITED STATES

## Abstract

Apicomplexan parasites invade host cells in an active process involving their ability to move by gliding motility. While the acto-myosin system of the parasite plays a crucial role in the formation and release of attachment sites during this process, there are still open questions regarding the involvement of other mechanisms in parasite motility. In many eukaryotes, a secretory-endocytic cycle leads to the recycling of receptors (integrins), necessary to form attachment sites, regulation of surface area during motility, and generation of retrograde membrane flow. Here, we demonstrate that endocytosis operates during gliding motility in *Toxoplasma gondii* and appears to be crucial for the establishment of retrograde membrane flow, because inhibition of endocytosis blocks retrograde flow and motility. We demonstrate that extracellular parasites can efficiently incorporate exogenous material, such as labelled phospholipids, nanogold particles (NGPs), antibodies, and Concanavalin A (ConA). Using labelled phospholipids, we observed that the endocytic and secretory pathways of the parasite converge, and endocytosed lipids are subsequently secreted, demonstrating the operation of an endocytic-secretory cycle. Together our data consolidate previous findings, and we propose an additional model, working in parallel to the acto-myosin motor, that reconciles parasite motility with observations in other eukaryotes: an apicomplexan fountain-flow-model for parasite motility.

## Abstract 2

Motility is crucial for the life cycle of apicomplexan parasites; evaluation of membrane lipids and surface protein uptake in Toxoplasma gondii sheds new light on the role played by endocytosis in the gliding motility of this and other apicomplexans.

## Introduction

The intracellular protozoan parasite *Toxoplasma gondii* infects nearly 2 billion people globally. This apicomplexan can cause severe disease in immunocompromised people and can lead to miscarriage or malformation of the foetus in pregnant women [1]. During the acute phase of infection, the tachyzoite rapidly replicates inside the host cell within a specialised compartment, the parasitophorous vacuole, which itself is formed during active invasion [2].

Like all apicomplexans, *T*. *gondii* invades host cells in an active process involving both the parasite’s ability to move by gliding motility and invasion factors derived from the unique secretory organelles localised at the parasite’s apical pole (micronemes and rhoptries) [3,4]. According to the linear motor model, micronemal transmembrane proteins are secreted at the apical tip of the parasite and act as force transmitters by interacting with both the substrate at the surface and the acto-myosin system of the parasite. While this motor system fulfils an important role for motility and invasion, recent studies highlighted that parasites are still capable of moving, despite the disruption of core components of the glideosome [5–11], leading to the question of whether, similar to other eukaryotes, parasites are able to use different motility mechanisms. In other eukaryotes, depending on the environments the cell has to move in, several (actin dependent and actin independent) motility mechanisms have been described, such as osmotic engines, blebbing motility, pressure-driven protrusions, or the well-understood amoeboid motility [12]. Apicomplexan parasites are masters of motility in different environments, allowing them to disseminate within and between their hosts, migrate through different tissues, and to invade virtually any host cell [3]. It would therefore not be surprising if apicomplexans, akin to other eukaryotes, can use more than one motility system, depending on the environment they need to move in. In fact, it is likely that these mechanisms can operate synergistically, which might explain observations that parasites are still able to move and invade, albeit at highly reduced rates, when the glideosome is disrupted [5–11].

During motility, most eukaryotic cells show a capping activity of surface ligands, which is dependent on actin, microtubules, and a secretory-endocytic cycle, leading to the establishment of a retrograde membrane flow [13,14]. A recent study on *Dictyostelium* provided direct evidence for the fluid flow model during cell migration [15]. This study demonstrated that, during migration of *Dictyostelium*, the membrane volume of the cell remains constant due to the occurrence of a secretory-endocytic cycle. This circulation follows a fountain-flow model, in which new membrane lipids are delivered to the anterior cell membrane, whereas excess membrane is recycled. Interestingly, in this study a direct relationship between cell migration and membrane turnover rate was observed, suggesting that the cells establish a fluid drive that contributes to the generation of force required for motility, as suggested previously [14]. Importantly, it appears that myosin and actin have only a supporting function in the establishment of the fluid drive, because treatment with actin- or myosin-disrupting drugs, such as latrunculin B or blebbistatin, did not significantly affect membrane movement [15]. Similarly, a recent study by O’Neill and colleagues [16] demonstrated that membrane flow itself can facilitate amoeboid migration of immune cells in diverse environments in the absence of specific molecular interactions with the surrounding medium.

In good agreement, the generation of retrograde membrane flow in apicomplexan parasites is not strictly dependent on parasite actin, as shown for *Plasmodium* sporozoites [11,17] and *Toxoplasma* tachyzoites [5]. In the case of *Plasmodium* [11,17], it was demonstrated that beads bound to the surface are translocated to the posterior at the same speed, even after disrupting actin, and parasites’ motility could be correlated to the generation of retrograde flow. However, if the bead is kept in place with a laser trap, disruption of F-actin results in reduced force pulling at the bead, leading to the conclusion that retrograde membrane flow and force production can be uncoupled. Similarly, in the case of *Toxoplasma*, disruption of actin results in significantly less association of beads to the surface of the parasite, but bound beads are translocated at a similar speed to the posterior of the parasite [5]. Together these data support an interpretation in which retrograde membrane flow can occur independently of parasite actin. However, strong attachment and force generation require the parasite’s glideosome under these conditions, leading to the question of how retrograde membrane flow is generated.

It is well accepted that motility of apicomplexans depends on the regulated secretion of the apically localised micronemes [4,18]. While this dependency was previously attributed to the secretion of surface ligands, such as the microneme protein 2 (MIC2), that are required as force transmitters, it is also possible that polarised secretion is required for the generation of retrograde membrane flow, akin to the fountain-flow model [15] and as previously suggested for *T*. *gondii* [5,19]. However, to date, it is not fully understood how apicomplexan parasites maintain a constant cell surface during motility by removing excess membrane deposited on the surface due to microneme secretion. While the shedding of membrane trails during motility [20–22] might contribute to a constant membrane content and cell surface, it appears likely that, as suggested by the fountain-flow model [15], excess membrane could also be internalised and recycled during motility.

Here, we set out to determine if extracellular parasites are capable of efficiently recycling membrane and taking up exogenous material via endocytosis. To date, uptake of exogenous material has been demonstrated during the intracellular stages of the parasite [23–26]. In other eukaryotes, endocytic processes play key roles in membrane dynamics, making it an important participant in cell motility [27,28]. Endocytosis can occur via different mechanisms and is roughly defined as clathrin-dependent endocytosis (CDE) or clathrin-independent endocytosis (CIE) [29]. Apicomplexan genomes lack many factors known to be involved in the endocytic system, such as endosomal sorting complexes required for transport (ESCRT) complexes, and previous reverse genetic analysis suggested that the remaining factors were repurposed to contribute to the biogenesis and maintenance of unique organelles, such as the inner membrane complex (IMC) or the secretory organelles [30–35].

Here, we demonstrate the implication of endocytosis in the maintenance of retrograde membrane flow and provide a link between this process and gliding motility, in good agreement with the fountain-flow model [14,15]. We demonstrate the capacity of extracellular tachyzoites to take up phospholipids, nanogold particles (NGPs), antibodies directed against parasite surface proteins, and Concanavalin A (ConA). Interestingly, endocytic uptake of material follows the known secretory pathway of the parasite, with accumulation of material in the rhoptries but also vacuolar-like compartment (VAC; or plantlike vacuole [PLV] [36,37]).

Together our data demonstrate the existence of a secretory-endocytic cycle during parasite motility that appears to be critical for motility and therefore fully supports the hypothesis that a fountain-flow model operates, as suggested for other motile eukaryotic cells [15].

## Results

### Fountain-flow model and evidence of endocytosis implication in *T*. *gondii* motility

**Fig 1:**
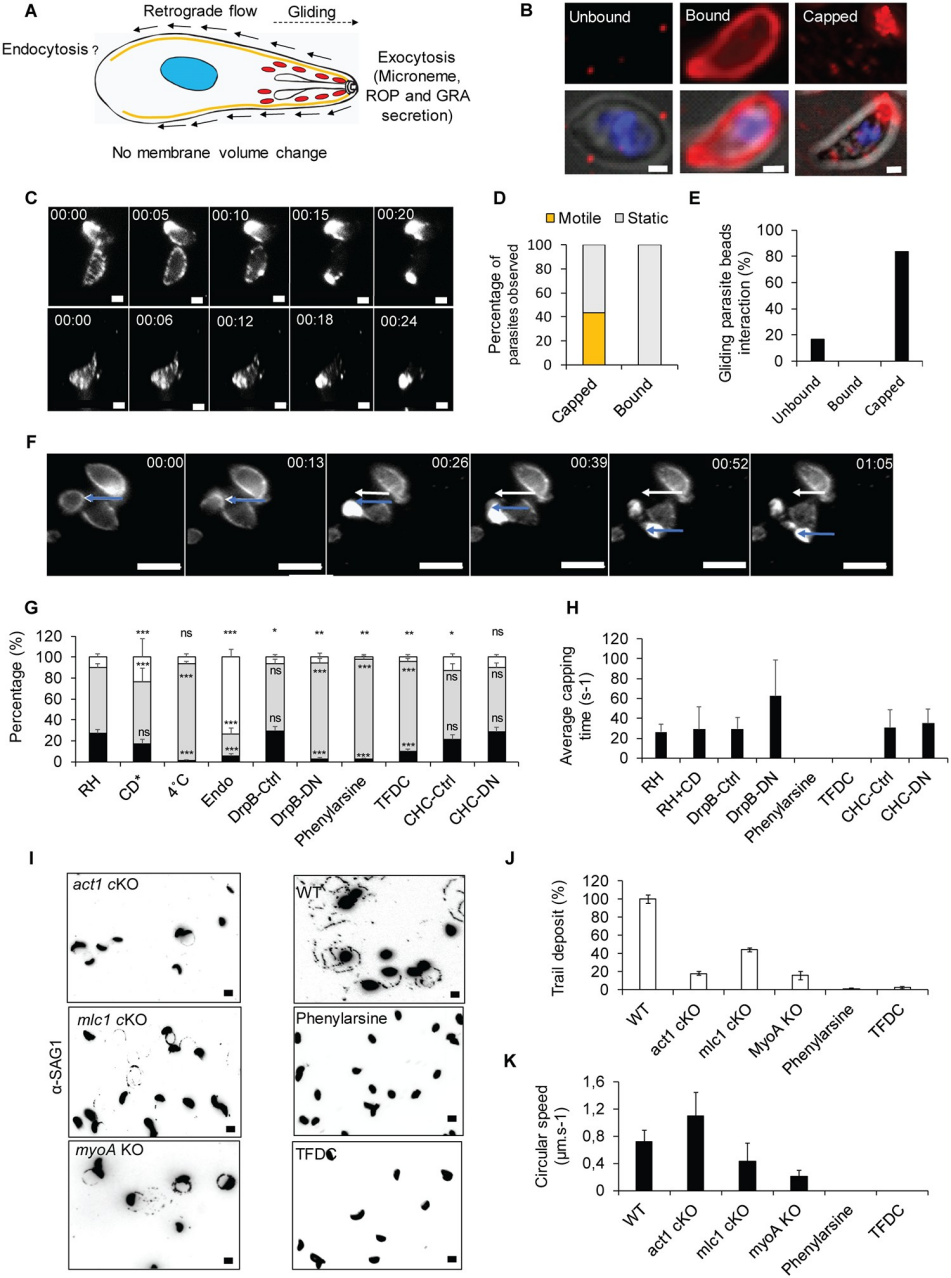
Evidence for involvement of endocytosis in *T*. *gondii* motility. (A) Fountain-flow model described for *Dictyostelium* by Takana and colleagues 2017 [13], applied to *T*. *gondii*. While it was demonstrated that motility depends on the apical secretion of micronemes, the role of endocytosis to ensure membrane balance and recycling is unclear. (B) Representative pictures of the three phenotypes observed in the bead translocation experiment. Unbound: parasite without beads, that either did not interact or lost its interaction with the beads; Bound: parasite with beads around the plasma membrane; Capped: parasite that translocated bound beads to its basal pole. Scale bar, 1 μm. (C) Time-lapse analysis of bead translocation. Parasites were incubated with latex beads. Capping was recorded by live microscopy to determine the average time required for capping. Scale bar, 1 μm. (D) Quantification of motility in capped and bound parasites. Capped: *n* = 175; Bound: *n* = 166. (E) Quantification of gliding parasites in combination with bead translocation. Only parasites with unbound (no beads) and capped beads (beads accumulated at the posterior end) were observed gliding. No instances in which beads were bound at the plasma membrane, but not capped, were observed; *n* = 38. (F) Time-lapse illustration of a parasite that translocated beads and initiates gliding motility. Scale bar, 5 μm. The white arrow indicates the initial position of the basal pole of the parasite. The blue arrow indicates the actual position of the basal pole of the parasite. Capping occurred within the first 26 seconds, and then the parasite initiated motility. (G) Quantification of bead translocation using indicated inhibitors or parasite mutants. Unbound (white), bound (grey), and capped (black). Parasites expressing dominant negative versions of DrpB-DN or CHC-DN were induced with 1 μM Shield-1, as described previously [31,33]. Inhibitors were used at the following concentrations: 0.5 μM CD, 10 μM phenyl arsine oxide, or 50 μM TFDC. Mean values of three independent assays are shown ± SD. ***p < 0.001 in a two-tailed Student *t* test compared with RH without inhibitors. *Data extracted from Whitelaw and colleagues, 2017 [4]. (H) Analysis of the average time required for capping under the conditions shown in (G). Analysis in the presence of endocytosis inhibitors was not possible, because no capping could be observed under these conditions. (I-K) Trail deposition assay of WT parasites compared with motor mutants (*act1* cKO, *mlc1* cKO, and *myoA* KO) and endocytosis inhibitors (phenylarsine and TFDC). (I) Representative pictures, scale bar, 5 μm. (J) Quantification of the trail deposition. Mean values of three independent assays are shown ± SD. (K) Analysis of average gliding speed. Mean values of three independent assays are shown ± SD. For each bar graph, the corresponding data can be found in S1 Data. CD, Cytochalasin D; CHC, clathrin heavy chain; cKO, conditional knockout; Ctrl, control non-induced; DN, dominant negative; DrpB, dynamin-related protein B; Endo, endo buffer; KO, knockout; TFDC, trifluoperazine dihydrochloride; WT, wild-type.

The fountain-flow model has been recently demonstrated to operate during eukaryotic cell motility, such as in *Dictyostelum discoideum* [15]. This model predicts the establishment of a retrograde membrane flow by localised secretion (at the anterior end of the cell), followed by endocytic recycling to ensure membrane balance. In this respect, apicomplexan parasites are a prime example of highly polarised cells, in which the micronemes are secreted at the apical tip during gliding (Fig 1A), which, in analogy to *D*. *discoideum*, should result in retrograde membrane flow.

To analyse retrograde membrane flow in *T*. *gondii* tachyzoites, we previously adapted a translocation assay [38] that follows the transport of fluorescent beads to the posterior end of the parasite. This ‘capping’ has been directly implicated in the motility of *T*. *gondii* and, importantly, can occur in the absence of the acto-myosin motor [5]. Three phenotypes are observable during this assay: ‘Unbound’ parasites without beads, ‘Bound’ parasites with beads distributed along the plasma membrane, and ‘Capped’ parasites that have translocated the bead to their basal pole (Fig 1B).

We performed live imaging of the capping process in the presence and absence of different inhibitors or proteins that interfere with the acto-myosin system, secretion, or endocytic processes in other eukaryotes (Fig 1C and S1 Video). We incubated parasites with 40-nm latex beads at 4 °C to allow binding of the beads to the parasites surface. Upon a temperature shift to 37 °C, capping occurs rapidly, and beads accumulate at the posterior pole of the parasite within approximately 25 seconds (Fig 1C). Importantly, we found that parasite gliding correlates with bead translocation (Fig 1D, 1E and 1F and S2 Video), because approximately 84% of motile parasites showed translocation, while approximately 16% of motile parasites did not have any beads on the surface (Fig 1E), indicating that no initial binding of the beads occurred or that beads were shed after translocation, as seen in S3 Video. Importantly, no gliding parasites were identified in which beads remained immobile while bound to the parasite plasma membrane (Fig 1D and 1E). Interestingly, parasites can translocate beads without moving, demonstrating that retrograde flow can occur in the absence of gliding, while gliding does not occur in the absence of retrograde flow.

The mechanism underlying parasite membrane balance is unknown and suggested to depend exclusively on membrane shedding and processing of micronemal transmembrane proteins [39]. We hypothesised that, akin to other eukaryotes, membrane balance is also ensured by endocytic recycling of excess membrane and proteins [15]. To determine if *T*. *gondii* retrograde flow could be dependent on a similar mechanism, we tested conditions that inhibit or alter secretion/exocytosis using established inhibitors of endocytosis or parasite strains, such as parasites expressing the dominant negative (DN) version of dynamin-related protein B (DrpB) [33], in which micronemes organelles are absent (Fig 1G).

Parasites incubated on ice bound beads (93% ± 3%), but no translocation was observed; a temperature shift to 37 °C resulted in translocation in approximately 27% of parasites. Interestingly, incubation of parasites in the presence of 0.5 μM Cytochalasin D (CD; a drug used to disrupt F-actin) did not result in significant reduction of bead translocation (Fig 1G), confirming that retrograde membrane flow can occur in the absence of a functional acto-myosin system, as reported previously [5]. In sharp contrast, abrogation of microneme secretion, either by incubation of parasites in endo buffer [2] or depletion of the microneme organelles (by inducing DrpB-DN with shield in intracellular parasites for a full lytic cycle before experimentation), abolishes bead translocation, demonstrating that retrograde flow depends on polarised secretion, as predicted by the fountain-flow model (Fig 1A; [15]). To evaluate if capping could depend on endocytosis, we used well-established inhibitors of endocytosis, such as Phenylarsine oxide [40] and trifluoroperazine [41], as well as a DN strain for clathrin heavy chain (CHC-DN) [31]. While the endocytosis inhibitors abrogated capping (2% ± 1% and 10% ± 1% capping, respectively), expression of DN CHC did not result in significant reduction of capping (−Shield: 21% ± 3%, +Shield: 29% ± 3% capping). Together, these results suggest that retrograde membrane flow depends on an endocytic mechanism, which might be a form of CIE. In good agreement, to date, no clathrin-coated vesicles have been identified at the parasite surface, and a previous study did not implicate CHC in endocytosis in *T*. *gondii* [31].

Next, we determined the average time required for capping under the same conditions as above (Fig 1H). In control parasites, capping occurs within 26 ± 7 seconds. Interference of F-actin using CD did not have a significant effect on capping time (29 ± 22 seconds). In contrast, interfering with secretion of micronemes by expression of DrpB-DN resulted in a significantly longer capping time of 63 ± 36 seconds (in the few instances when capping could be observed). Incubation of parasites with endocytosis inhibitors (phenylarsine or trifluorperasine) resulted in a complete block of capping. No difference was observed upon expression of CHC-DN (−Shield: 31 ± 18 seconds versus +Shield: 35 ± 14 seconds), again suggesting CIE.

When motility was analysed using the same conditions, we found a clear correlation between capping and motility. We confirmed previous findings [5,42], demonstrating that interference with the acto-myosin system of the parasite results in significantly reduced overall gliding motility (Fig 1I and 1K). Importantly, the few parasites still capable of gliding did so at similar speeds as control parasites (Fig 1J), as described previously [5]. In contrast, conditions that resulted in less and/or slower capping resulted in both fewer parasites capable of initiating gliding motility (Fig 1I and 1J) and parasites moving significantly slower (Fig 1K), when compared with controls.

Together these data strongly suggest a link between parasite motility and retrograde membrane flow, which appears to rely on secretion and endocytosis, as proposed by the fountain-flow model (Fig 1A).

### Extracellular *T*. *gondii* tachyzoites can take up labelled lipids

**Fig 2:**
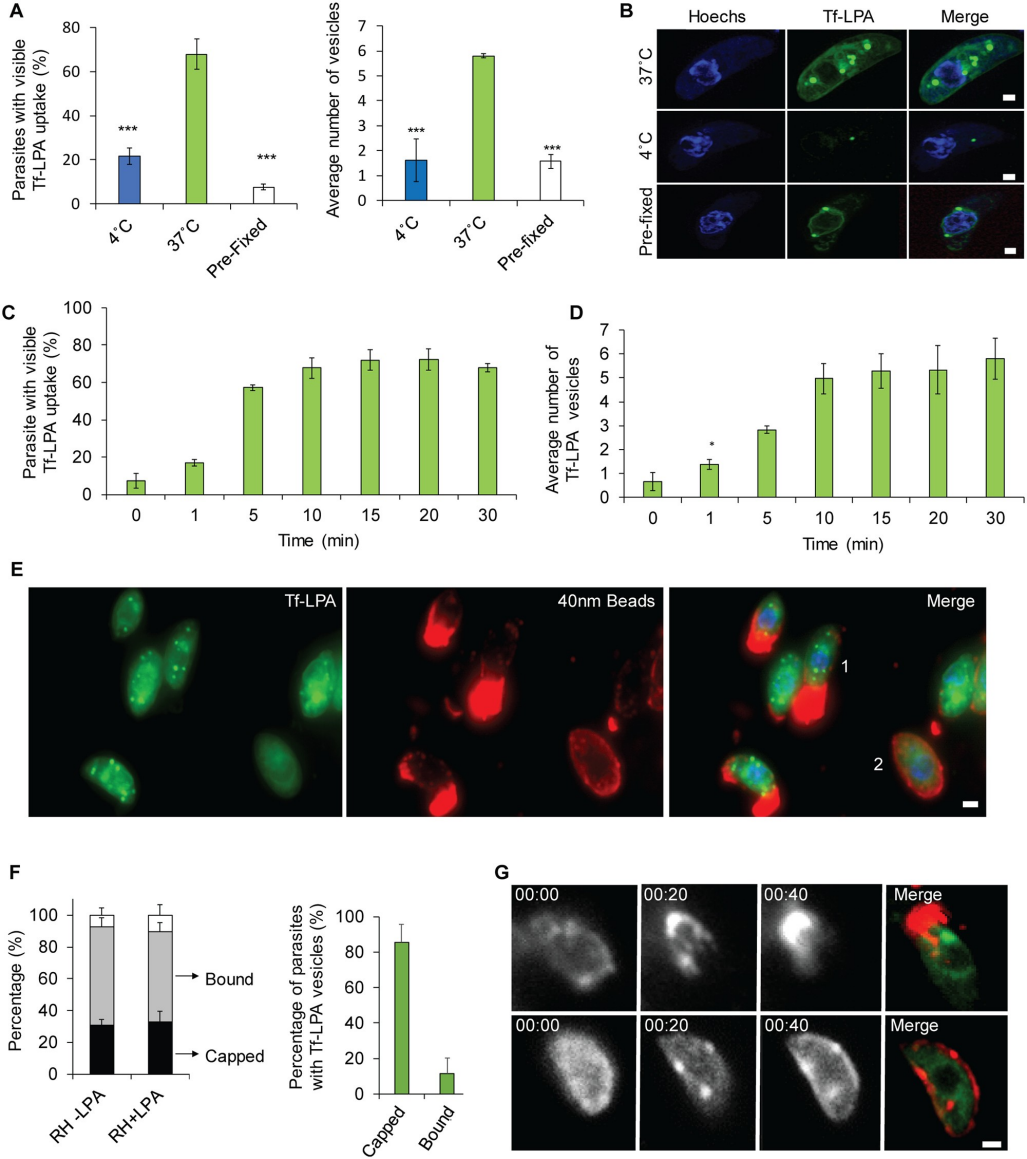
A link between retrograde flow and phospholipid uptake. (A-B) Uptake of phospholipids: Tf-LPA was analysed at 37 °C and 4 °C. Pre-fixed parasites with PFA were also incubated with Tf-LPA at 37 °C. Incubation at 37 °C demonstrates a strong uptake with vesicle formation. Uptake is inhibited at 4 °C or when the parasites were fixed prior to incubation. (A) Left panel: percentage of Tf-LPA–positive parasites in each of the tested conditions. Right panel: average number of Tf-LPA–positive vesicles per parasite in each of the tested conditions. Mean values of three independent assays are shown ± SD. ***p < 0.001 obtained in a two-tailed Student *t* test when comparing Tf-LPA at 37 °C versus 4 °C and live parasites plus Tf-LPA versus fixed parasites plus Tf-LPA at 37 °C. (B) Example images obtained for the quantification as shown in (A). Scale bar, 1 μm. (C-D) Dynamics of uptake were analysed in a time point assay from 0 to 30 minutes after addition of Tf-LPA. (C) Average number of parasites that show overall uptake and (D) average number of vesicles per positive parasite were determined. Mean values of three independent assays are shown ± SD. (E-G) Association of retrograde flow and endocytosis was analysing by adding Tf-LPA during the bead translocation experiment. (E) Representative picture of capping and Tf-LPA uptake. An example of each phenotype can be observed: capped (1); bound (2). Scale bar, 1 μm. (F): Left panel: quantification of the percentage of capped and bound parasites that also took up Tf-LPA; right panel: percentage of capped or bound beads only in parasites showing Tf-LPA uptake. Most parasites that translocated beads also endocytosed Tf-LPA. Mean values of three independent assays are shown ± SD. (G): Time-lapse analysis of bead translocation in the presence of Tf-LPA. Examples of a parasite in which translocation occurred (top) and did not occur (bottom) are shown. Scale bar, 1 μm. For each bar graph, the corresponding data can be found in S1 Data. PFA, paraformaldehyde; Tf-LPA, Top-Fluor lysophosphatidic acid.

To investigate if membrane balance could be, akin to other eukaryotes, maintained by endocytic recycling of excess membrane [15], we assessed the capacity of *T*. *gondii* to take up different fluorescent lipids, such as FM-dyes, Cell-Mask, Top-Fluor lysophosphatidyl choline (Tf-LPC), Top-Fluor lysophosphatidic acid (Tf-LPA), or the fluorophore Bodipy (Fig 2A and S1 Fig). In all cases, we observed efficient uptake of the dyes/lipids, with a clear difference between 4 and 37 °C. In cases of FM-dyes and Cell-Mask, the signals obtained appeared rather diffuse within the parasite (S1 Fig). In contrast, uptake of labelled phospholipids was characterised by the occurrence of sharp, discernible vesicles inside the parasite that might be associated with the parasite’s secretory system (Fig 2A and 2B and S1C Fig). These lipids were taken up at comparable rates (Bodipy: 83% ± 10%, Tf-LPC: 66% ± 9%, Tf-LPA: 68% ± 6%; S1 Fig), and uptake only occurred when parasites were incubated at 37 °C, while no similar uptake was observed at 4 °C or when dead parasites were incubated with these lipids (Fig 2A and 2B). No toxic effect or alteration in invasion, replication, or parasite morphology was observed in parasites incubated with Tf-LPA (S2 Fig). Together, these data demonstrate an active uptake of phospholipids in the majority of extracellular parasites. Next, we analysed the uptake of Tf-LPA over time (Fig 2C and 2D). Interestingly, intracellular Tf-LPA can be detected rapidly (from 17% ± 2% after 1 minute to 68% ± 2% 30 minintes after addition; Fig 2C). Furthermore, the average number of intracellular vesicles increases over time, from approximately 2 at 1 minute to approximately 6 at 30 minutes after addition of Tf-LPA (Fig 2D).

As described above, we were able to observe a link between the capping of beads and motility. To determine if a similar correlation between retrograde membrane flow and lipid uptake exists, we co-incubated parasites with 40-nm beads and Tf-LPA (Fig 2E, 2F and 2G). The addition of lipids did not significantly increase the overall capping activity of *T*. *gondii* (percentage of capped parasites: −Tf-LPA, 31% ± 6% versus +Tf-LPA, 33% ± 6%; Fig 2F). Interestingly, we observed a strong correlation between uptake of Tf-LPA and bead translocation, because almost all capped parasites (86% ± 9%) also presented Tf-LPA–positive vesicles, as illustrated in Fig 2E. In contrast, parasites that only bound latex beads to their surface did not show a high percentage of Tf-LPA uptake (12% ± 8%, Fig 2E and 2F). This correlation between capping and Tf-LPA uptake was also demonstrated using live microscopy (Fig 2G and S4 Video). Taken together, these data clearly illustrate that *T*. *gondii* is able to take up labelled lipids, which accumulate within vesicles. Moreover, this uptake correlates with the generation of retrograde flow.

### Lipid uptake converges with the secretory pathway

**Fig 3:**
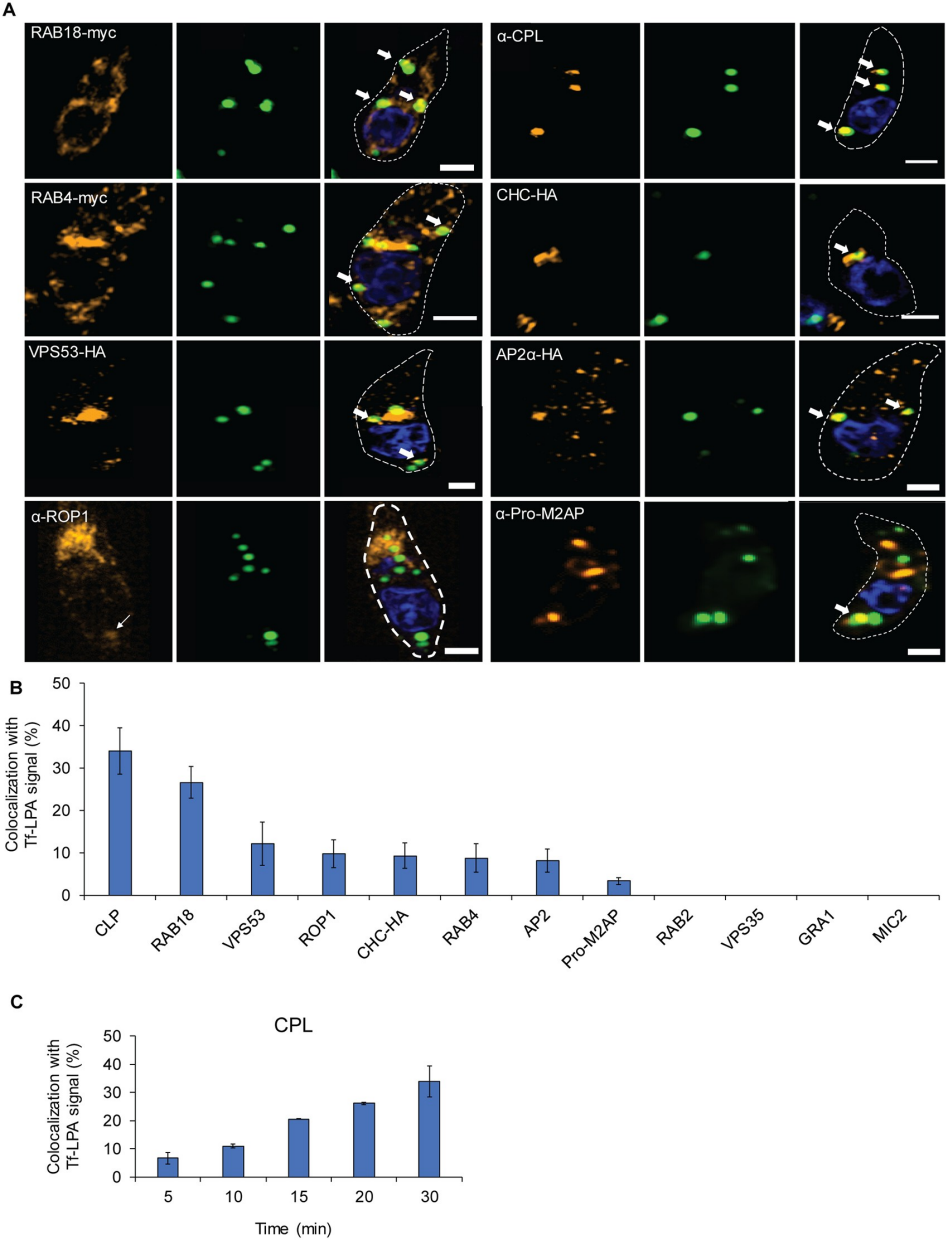
Endocytosed lipids traverse the secretory system. (A-B) Tf-LPA vesicle localisation was determined using indicated parasite strains expressing markers for the parasite trafficking system: dd-RAB18-myc: ER, dd-RAB4-myc: Golgi, VPS53-HA: TGN, VPS35-HA: Retromer/ELC, CHC-HA: Golgi, TGN, AP2α-HA parasites [31,32,43,44]. Antibodies against the VAC (α-CPL [37]), rhoptries (α-ROP1), micronemes (α-MIC2), and dense granules (α-GRA1) were used after fixation on RH parasites. (A) Representative pictures of colocalisations are shown (see also S4 Fig); scale bar, 1 μm. (B) Quantification of colocalisations. Mean values of three independent assays are shown ± SD. (C) Accumulation of Tf-LPA in the VAC over time. Percentage of colocalisation between Tf-LPA and CPL was determined from 0 to 30 minutes after Tf-LPA addition. Mean values of three independent assays are shown ± SD. For each bar graph, the corresponding data can be found in S1 Data. CHC, clathrin heavy chain; CPL, cathepsin L; dd, destabilisation domain; ELC, endosome-like compartment; ER, endoplasmic reticulum; Tf-LPA, Top-Fluor lysophosphatidic acid; TGN, *Trans*-Golgi network; VAC, vacuolar-like compartment; VPS, vacuolar protein sorting-associated protein.

We were interested in defining the pathway followed by incorporated lipids in detail. To this end, we performed colocalisation assays of Tf-LPA–positive vesicular structures with previously described markers of the parasite secretory pathway (S1 Table 1, Fig 3, and S3 Fig). The highest accumulation of Tf-LPA could be observed in the VAC (31% ± 9%), a plantlike vacuole in the parasite [36,37]. The second-highest colocalisation was observed with RAB18 (27 ± 4), a marker of the endoplasmic reticulum (ER) [32]. Tf-LPA also accumulated, to a lesser extent, with other organelles such as endosomes (vacuolar protein sorting-associated protein [VPS]53: 12% ± 5%), Golgi (Rab4: 9% ± 3% and CHC: 9% ± 3%), rhoptries (ROP1: 10% ± 3%), and the endosome-like compartment (ELC) (pro-M2AP 3% ± 1%). No colocalisation was observed with Rab2 (ER), VPS35 (ELC), MIC2 (micronemes), and GRA1 (dense granules; S3 Fig). With a time course analysis of the colocalisation rate with the VAC, we observe that it is increased over time (Fig 3C), suggesting a trafficking of labelled vesicles to the VAC as described for intracellular parasites [23]. Together, these data demonstrate that Tf-LPA accumulates in vesicles that are trafficked through the secretory pathway, with a certain accumulation in the VAC.

### A link between endocytosis and exocytosis of lipids

**Fig 4:**
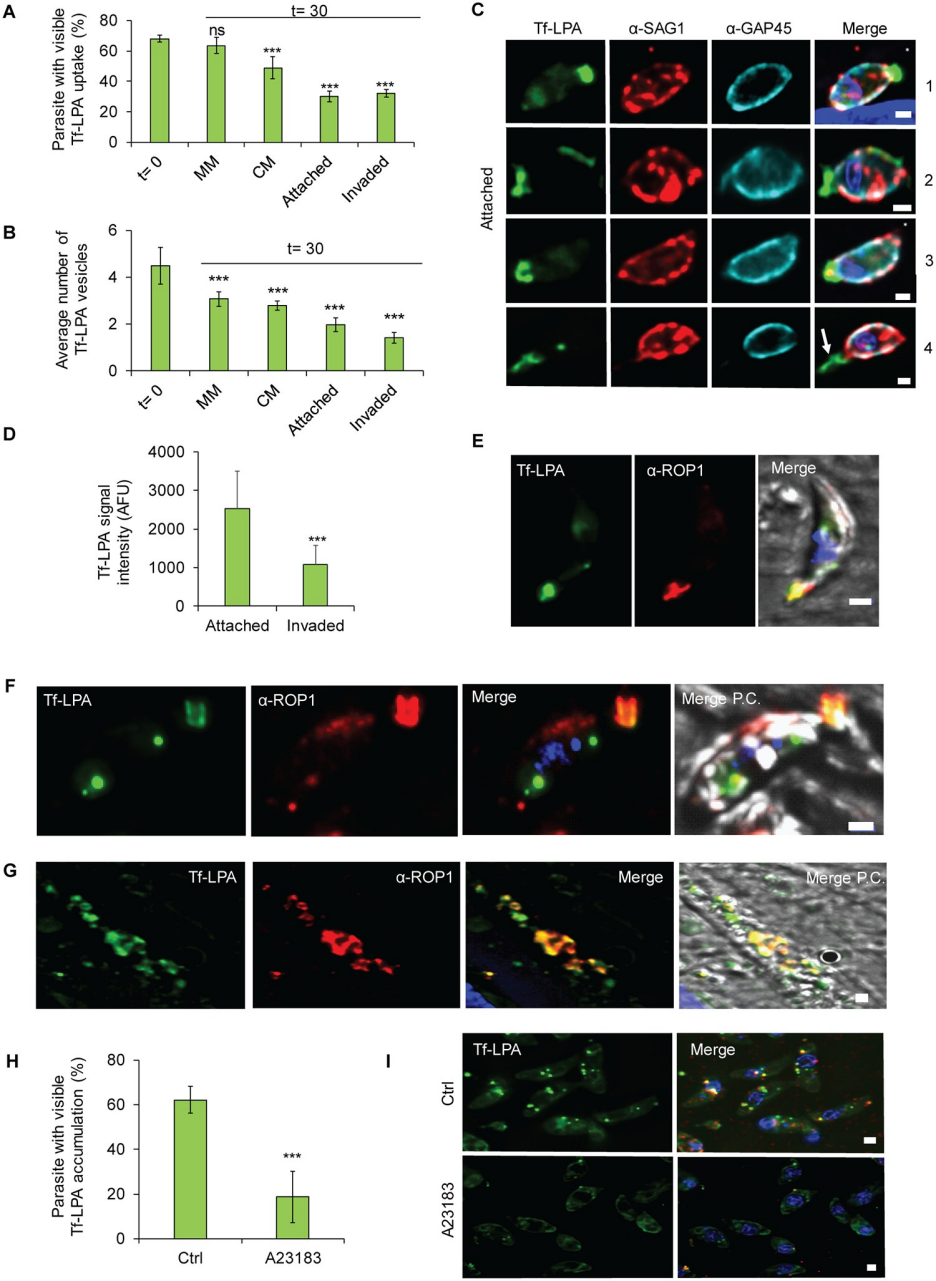
Tf-LPA follow an endocytic-exocytic pathway. (A-E) Exocytosis of Tf-LPA was evaluated 30 minutes after placing parasites under three conditions: MM, CM, and CM with host cells. In the latter case, invaded and attached parasites were analysed separately. A clear diminishing of the signal is observed over time in stimulating conditions, demonstrating secretion of previously endocytosed Tf-LPA. (A) Percentage of Tf-LPA–positive parasites and (B) average number of vesicles were calculated. Mean values of three independent assays are shown ± SD. ***p < 0.001 in a two-tailed Student *t* test. (C) Illustration of parasites transferred onto host cells. SAG1 staining (prior to permeabilisation) was used to differentiate intra- from extracellular parasites. Scale bar, 1 μm. From top to bottom: attached parasites: (1) parasite with apical accumulation of Tf-LPA, (2) parasite with partial membrane labelling with Tf-LPA, (3) parasite with Tf-LPA accumulated at the basal pole, (4) parasite with Tf-LPA left in the trail. Asterisk (*) indicates the apical pole of the parasite. (D) Quantification of Tf-LPA signal intensity between attached and invaded parasites. (E) IFA using α-ROP1 confirming the apical presence of Tf-LPA. (F, G) Secretion of Tf-LPA inside evacuoles was tested. Colocalisation between Tf-LPA (green) and α-ROP1 (red), as observed in evacuoles. (H-I) Impact of secretion stimulation on the accumulation of Tf-LPA vesicles. Exocytosis was stimulated using the calcium ionophore A23187. (H) The uptake Tf-LPA under the presence or absence of A23187 was quantified. Mean values of three independent assays are shown ± SD. ***p < 0.001 in a two-tailed Student *t* test. (I) Representative pictures of both uptake conditions. Scale bar, 1 μm. For each bar graph, the corresponding data can be found in S1 Data. AFU, arbitrary fluorescent unit; CM, complete media; Ctrl, control; IFA, immunofluorescence assay; MM, minimal media; P.C., phase contrast; Tf-LPA, Top-Fluor lysophosphatidic acid.

If a secretory-endocytic cycle operates within the parasite, it is possible that material entered via the endocytic route could be recycled and secreted. To test this hypothesis, parasites were pretreated with Tf-LPA for 30 minutes before excess material was washed away and parasites transferred to new dishes containing minimal media (MM), complete media (CM), or host cells with CM for 30 minutes before fixation (Fig 4A, 4B and 4C and S4 Fig). In MM, the percentage of parasites containing Tf-LPA was as high as before washing (*t* = 0 68% ± 2% versus MM 64% ± 5%). In contrast, when the parasites were placed in CM or in the presence of host cells, a drastic reduction was detected both in the percentage of parasites containing Tf-LPA (*t* = 0, 68% ± 2% versus CM 49% ± 7% versus invaded 32% ± 2% or attached parasites 30% ± 3%, Fig 4A), as well as the average number of vesicles per positive parasites (Fig 4B).

Furthermore, under these conditions a different distribution of Tf-LPA became evident (Fig 4C and 4E and S3 Fig), in which LPA localised to the apical pole, as confirmed by colocalisation with α-ROP1 (Fig 4E), the basal end of the parasite and the parasite surface (Fig 4C1-3). Tf-LPA was also observed in trails when parasites were incubated with host cells, suggesting secretion of the Tf-LPA–positive vesicles (Fig 4C-4). The hypothesis of secretion of Tf-LPA was also supported by a decreased intensity of the Tf-LPA signal in attached and invaded parasites (Fig 4D).

To further evaluate if Tf-LPA could be secreted, we tested the presence of Tf-LPA in evacuoles [45]. Indeed, LPA-positive evacuoles could be identified, as evidenced by costaining with ROP1 antibodies, when parasites were allowed to attach to host cells in the presence of CD ([45], Fig 4F and 4G), demonstrating that internalised Tf-LPA can be secreted.

Finally, to investigate secretion of internalised Tf-LPA in more detail, we tested the accumulation of Tf-LPA in vesicles in the presence or absence of 2 μM calcium ionophore A23187, which triggers microneme secretion (Fig 4I and 4J). The addition of calcium ionophore led to a significant reduction of Tf-LPA accumulation inside the parasites. Taken together, these data illustrate that labelled lipids follow an endocytosis-secretion cycle.

### Endocytosis of exogenous material

**Fig 5:**
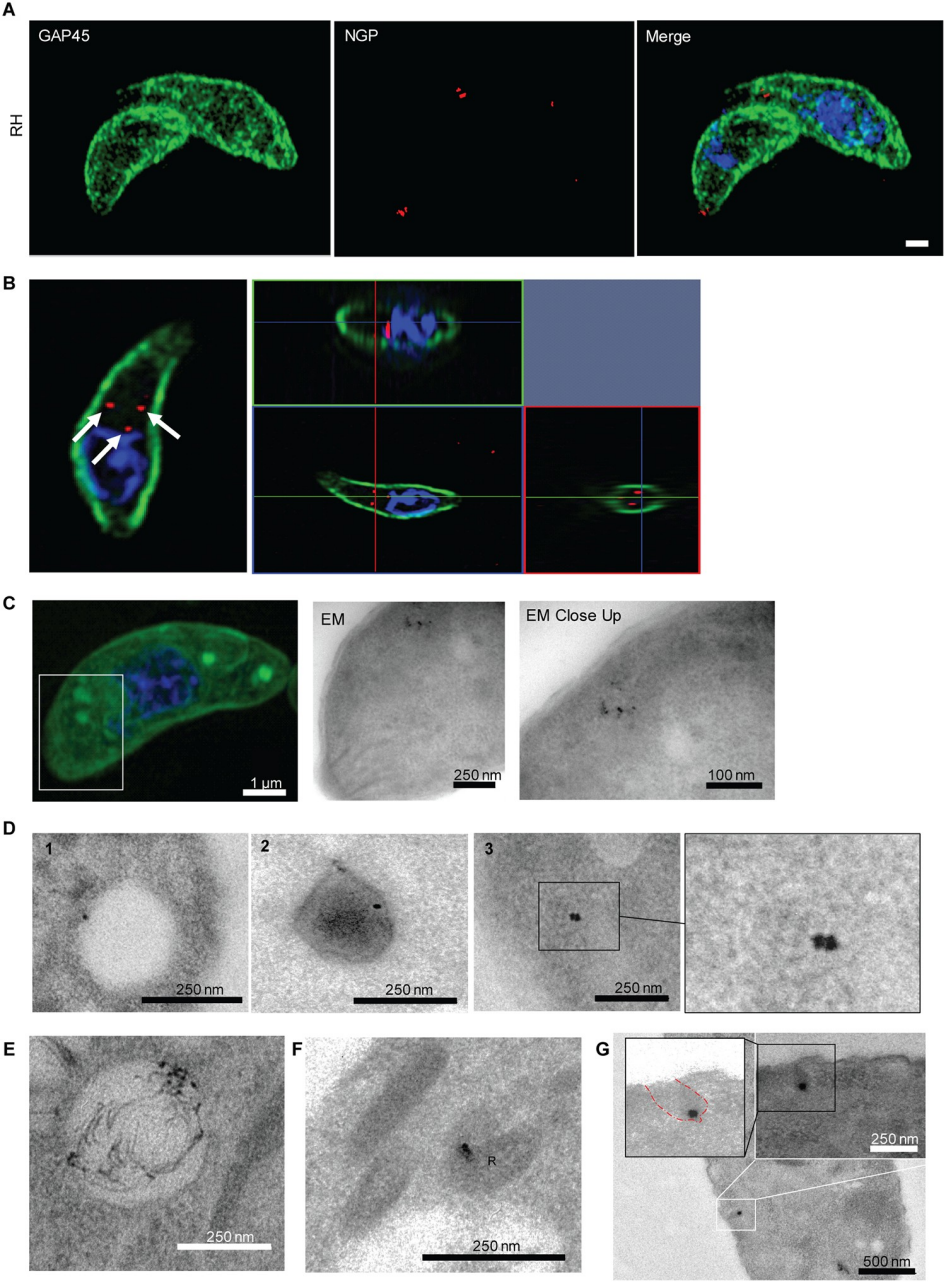
NGPs are taken up by *T*. *gondii*. (A-B) Uptake of NGPs was tested on RH- and RH Tf-LPA–treated parasites with 10-μm Cy5 conjugated gold beads. Parasites were imaged using 3D-SIM microscopy. NGPs were found to accumulate below GAP45, as illustrated by either maximum intensity projection (A) or ortho-view (B). Scale bar, 1 μm. (C) CLEM imaging of Tf-LPA and NGP uptake. After the uptake experiment, parasites were imaged using super-resolution microscopy (scale bar, 1 μm) before imaging by EM to evaluate if NGPs and Tf-LPA are inside the same vesicles. White square: close-up illustrated by the first TEM image; black square, TEM close-up. (D-G) TEM localisation of NGP. Scale bar size is indicated on the images. (D) Representative images of the different types of vesicles observed: (1) large translucent vesicles (300–500 nm), (2) medium-sized dense vesicles (215–375 nm), and (3) small vesicles (80–200 nm). (E) Localisation of the NGPs in the VAC, (F) localisation of the NGPs in rhoptry bulbs (labelled with R) by TEM. (G) Image of a potential entry point as an invagination of the plasma membrane containing NGP. CLEM, correlative light and electron microscopy; EM, electron microscopy; NGP, nanogold particle; TEM, transmission electron microscopy; Tf-LPA, Top-Fluor lysophosphatidic acid; VAC, vacuolar-like compartment; 3D-SIM, three-dimensional structure illumination microscopy.

After investigating lipid uptake, we wondered if extracellular parasites, like intracellular parasites [23], are capable of taking up bulkier material. In the absence of well-established protein markers for endocytosis, we first decided to analyse the uptake of 10-nm NGPs that are regularly used to analyse endocytosis in other eukaryotes [46]. These inert particles are dragged passively with the membrane flow but will not be specifically trafficked to certain destinations, once inside the parasite. When wild-type parasites were incubated with NGPs, it was possible to detect NGP uptake in vesicular structures (Fig 5A and 5B and S5A Fig) that appeared to be similar to the structures observed with Tf-LPA (Fig 5B, S4 Fig and S5 Video). To investigate whether NGPs and Tf-LPA colocalise in the same compartments, we performed correlative light and electron microscopy (CLEM). Parasites were incubated with Tf-LPA and NGPs for 30 minutes before fixation and indeed we observed NGPs in Tf-LPA–positive vesicles (Fig 5C). As observed in the case of Tf-LPA vesicles, NGPs were found in different locations within extracellular tachyzoites, because electron microscopy demonstrated that NGPs accumulated within vesicles of varied density (Fig 5D). They were found in at least three types of vesicles: large translucent vesicles (300–500 nm, Fig 5D panel 1), medium-sized dense vesicles (215–375 nm, Fig 5D panel 2), and small vesicles (80–200 nm, Fig 5D panel 3 and 3′). In good agreement with the Tf-LPA colocalisation experiments, NGPs were observed inside the VAC (Fig 5E) but also inside the rhoptries, supporting our observation that internalised Tf-LPA could be detected in evacuoles (Fig 4F and 4G). This indicates that internalised membranes are recycled towards the secretory organelles (Fig 5F). In contrast, NGPs were never seen in dense granules or micronemes. We also identified potential invaginations at the surface of the parasite that contain NGPs, probably representing the point of uptake (Fig 5G). These structures are delineated by the plasma membrane, demonstrating that NGPs are actively taken up in an endocytic-like process. Importantly, these invaginations are not electron dense, and a classical clathrin cage could never be detected, suggesting a CIE mechanism (Fig 5G).

**Fig 6:**
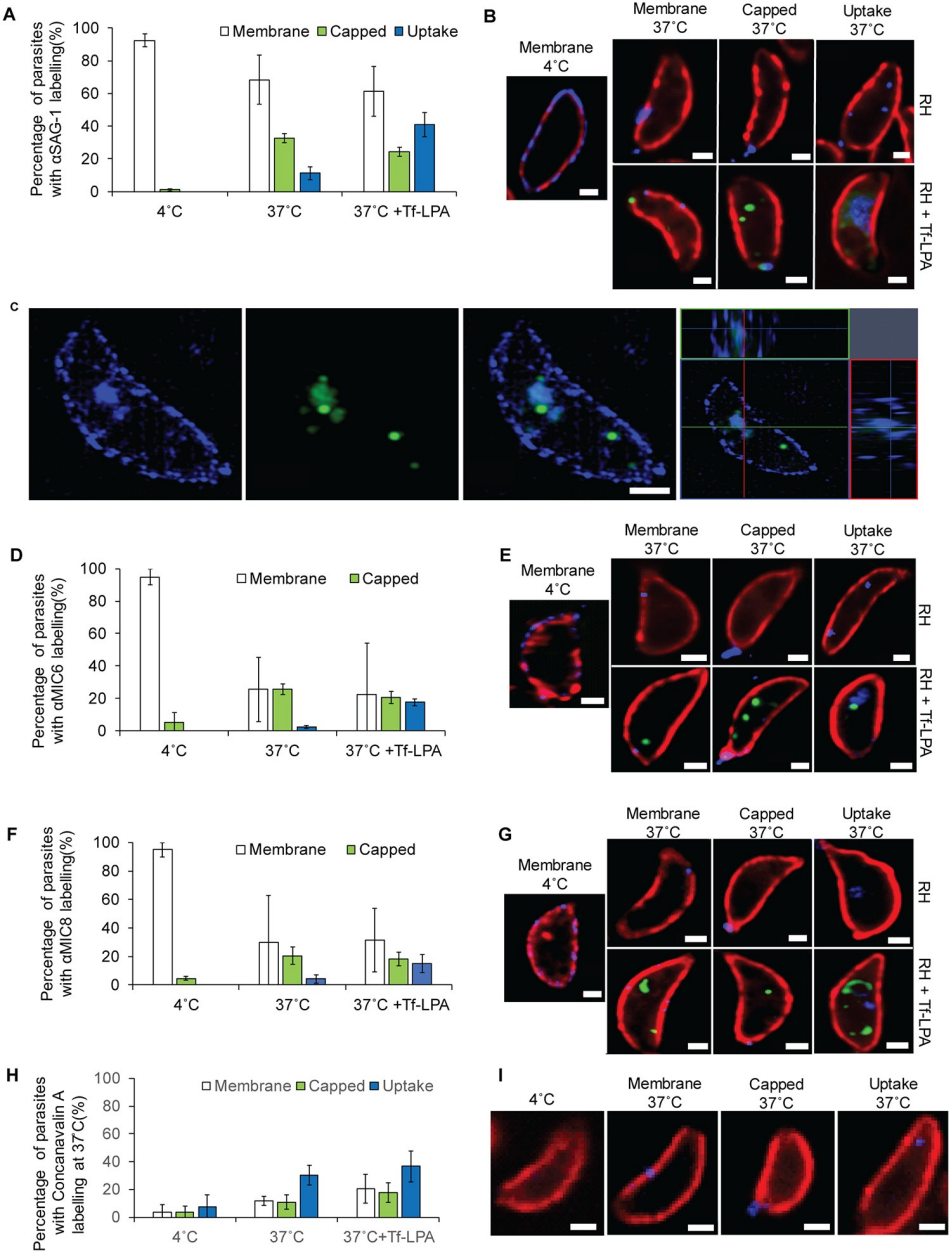
Uptake of surface proteins by *T*. *gondii* extracellular tachyzoites. (A-C) Uptake of SAG1 was analysed by labelling live parasite with αSAG1 and secondary antibodies at 4 °C before an additional incubation at 4 °C, 37 °C, or 37 °C with Tf-LPA for 30 minutes. (A) Quantification of the uptake of αSAG1 after 30 minutes. Parasites were divided into three categories: Membrane labelling, Capped, and Uptake. Mean values of three independent assays are shown ± SD. (B) Representative pictures of the αSAG1 labelling in indicated conditions. Blue, αSAG1; red, αGAP45; green, Tf-LPA. In RH, clear αSAG1 signal could be observed below GAP45. Scale bar, 1 μm. (C) Uptake of αSAG1 and Tf-LPA imaged by 3D-SIM microscopy. αSAG1 (blue); Tf-LPA (green). Scale bar, 1 μm. (D-E) MIC6 uptake: similar experiment to that for SAG1 uptake was performed for MIC6. (D) Quantification of parasites with membrane labelling, capped, and MIC6 uptake at 4°C, 37°C, or 37°C with Tf-LPA. Mean values of three independent assays are shown ± SD. (E) Representative pictures of MIC6 labelling after 30 minutes in the indicated conditions. Blue, αMIC6; red, αSAG1; green, Tf-LPA. Scale bar, 1 μm. (F-G) MIC8 uptake: conditions were analogous to MIC6 and SAG1. (F) Quantification of parasites with membrane labelling, capped, and MIC8 uptake at 4°C, 37°C, or 37°C with Tf-LPA. Mean values of three independent assays are shown ± SD. (G) Representative pictures of the αMIC8 labelling after 30 minutes in the different tested conditions. Blue, αMIC8; red, αSAG1; green, Tf-LPA. Scale bar, 1 μm. (H-I) ConA uptake was measured by the addition of Alexa-350 conjugated ConA to parasites at 4 °C and performing a temperature shift. (H) Quantification of parasites with membrane labelling, capped, and uptake of ConA at 37 °C. Mean values of three independent assays are shown ± SD. (G) Representative pictures of the ConA labelling after 30 minutes. Blue, ConA; Red, αGAP45. Uptake of ConA occurred more frequently than capping of ConA labelling. Scale bar, 1 μm. For each bar graph, the corresponding data can be found in S1 Data. ConA, Concanavalin A; Tf-LPA, Top-Fluor lysophosphatidic acid; 3D-SIM, three-dimensional structure illumination microscopy.

While the inert nature of NGPs is advantageous to visualise uptake using electron microscopy, they do not represent a physiological substrate for endocytosis. Therefore, we were interested if surface protein and other external material is taken up by the parasite in a similar way. We analysed the internalisation of the major surface antigen 1 (SAG1) and microneme proteins 6 and 8 (MIC6 and MIC8 present in different subsets of micronemes [32]). We incubated extracellular parasites in the presence of α-SAG1 at 4 °C and then coupled them directly with the secondary antibodies before placing the parasites at 37 °C. After 30 minutes, parasites were fixed and analysed for uptake of the antibodies. Incubation of parasites at 4 °C for 30 minutes or direct fixation after labelling showed no visible uptake (Fig 6A and 6B), with the majority of parasites exhibiting a labelling of the plasma membrane (92% ± 4%). In contrast, upon treatment of parasites for 30 minutes at 37 °C, we observed three different conditions (Fig 6A and 6B): (1) membrane labelling: antibodies were at the plasma membrane and/or can be concentrated at different areas of the plasma membrane other than the basal pole; (2) capped: antibodies accumulated at the basal pole of the parasite or are found in trails; (3) uptake: antibodies are detected inside the parasite, demonstrating their uptake. Membrane labelling was predominantly observed, followed by capping (33% ± 3%). In contrast, endocytosis could only be observed in 11% ± 4% of the parasites. It was noticed that some parasites presented two or three of the described labelling at the same time (S5B Fig). Interestingly, treatment of parasites with Tf-LPA leads to a significant increase in the internalisation of α-SAG1 (from 11% ± 4% to 41% ± 8%), concomitant with a reduction of the capped signal (from 33% ± 4% to 24% ± 3%) (Fig 6A and 6C and S5C Fig). Importantly, internalised SAG1 colocalises with Tf-LPA (approximately 90%), but not in the classical, very bright vesicles as illustrated in Fig 6C, indicating that it follows the same pathway or that both pathways are convergent.

Next, we performed analogous experiments to analyse the uptake of the micronemal proteins MIC6 (Fig 6D and 6E and S5D Fig) and MIC8 (Fig 6F and 6G and S5E Fig). Because micronemal proteins are stored within their organelles, we stimulated their secretion using calcium ionophore A23187 prior to shifting parasites to 4 °C and addition of the respective antibodies. Interestingly, the behaviour of both MIC6 and MIC8 appears to be almost identical (Fig 6D–6G), although they were previously shown to be present in different subsets of micronemes [32]. Upon incubation of parasites at 37 °C, the majority of parasites lost their antibody staining, relative to SAG1 or the 4 °C control, indicating that micronemal proteins (or micronemal proteins bound by an antibody) are efficiently removed from the surface, probably by the activity of rhomboid proteases [47,48]. Under these conditions, internalisation of MIC6 or MIC8 was only seen in a few parasites (approximately 5% and 2%, respectively). Similarly to the effect observed for SAG1 uptake, we found that addition of Tf-LPA significantly stimulated uptake of MIC6 and MIC8 (Fig 6D–6G).

Next, we wished to evaluate if endocytosis of surface proteins occurs in a more general manner. To that end, we used ConA, an excellent marker for membrane-bound endocytic activity in *Trypanosoma* [49]. When parasites were kept at 4 °C, no significant labelling could be detected. However, upon shifting to 37 °C, we observed membrane-bound, capped, and internalised ConA, similar to the situation seen for surface proteins (Fig 6H and 6I and S5F Fig). Internalised ConA could be detected in 30% ± 7% of parasites (Fig 6H and 6I). Interestingly, addition of Tf-LPA led only to a minor increase in ConA uptake (37% ± 11%), indicating that it is not a general stimulator of bulk endocytosis.

Taken together, these data show that surface proteins can be endocytosed by extracellular parasites and that addition of Tf-LPA stimulates the uptake of some membrane proteins. LPA is a phospholipid that is naturally present in serum [50] and has been reported to be an endocytosis stimulator in mammalian cells [51,52]. While it is possible that LPA acts as a physiological stimulator for endocytosis in *T*. *gondii*, future experiments are required to clarify the mechanism of stimulation.

**Fig 7:**
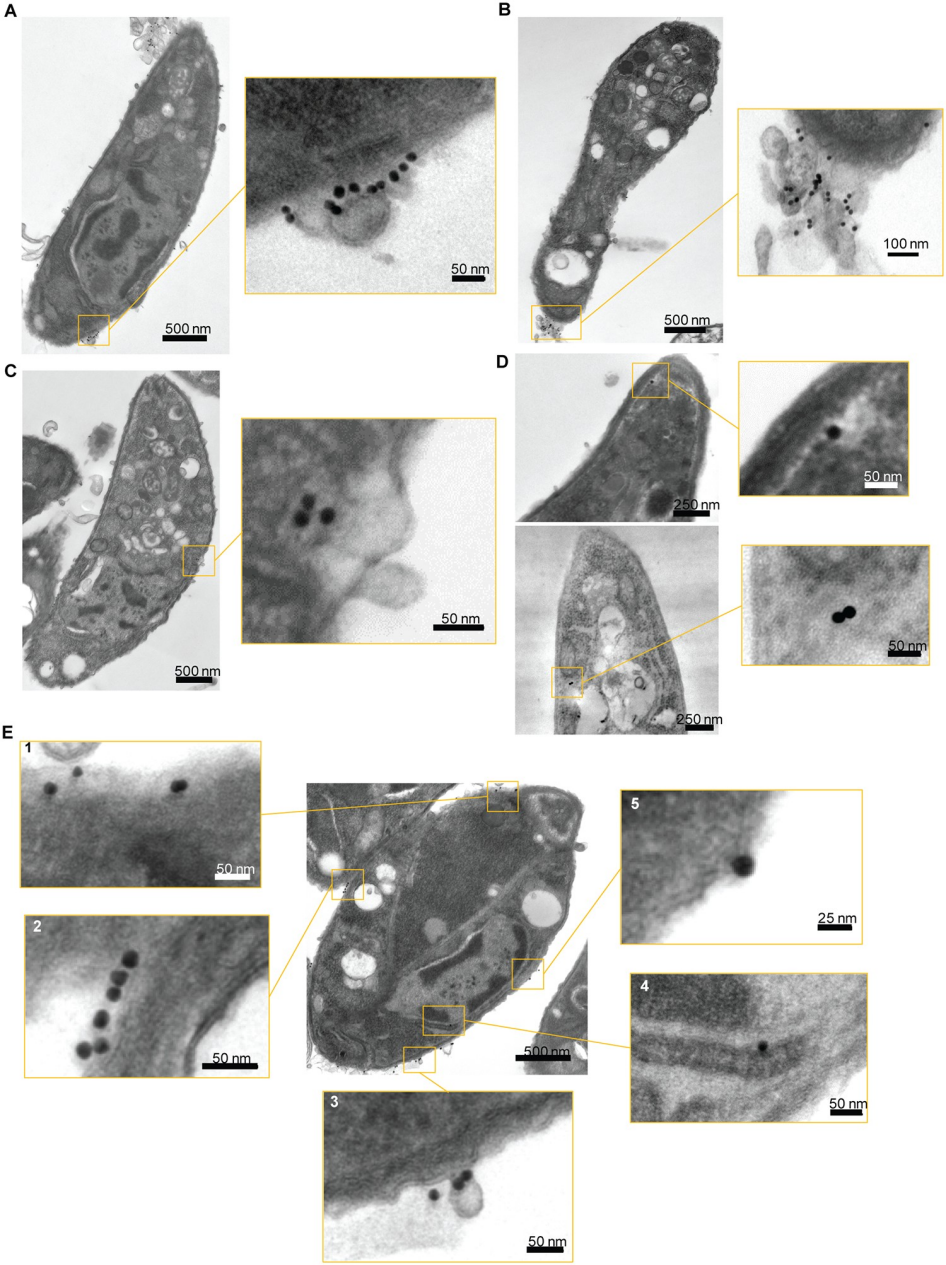
SAG1 uptake analysed by TEM. Uptake of SAG1 was also analysed by TEM. Secondary antibodies were coupled with 15-nm gold beads to label the parasites before incubation at 37 °C with Tf-LPA. (A-D) The three different labels observed by immunofluorescence assays (Fig 6) were also observed by TEM. (A) Parasite with αSAG1 bound to the membrane. (B) Parasite with αSAG1 capped. (C-D) Parasite with αSAG1 endocytosed at different locations. As for the NGP, the gold-coupled antibodies seem to be in translucent vesicles. (E) Transversal cut of a parasite, illustrating that labelling in different parts of the parasite can be observed at the same time even in TEM. Five close-up images are showing the different types of interactions. (1) Beads below the plasma membrane, (2) beads at the surface of the plasma membrane, (3) beads located on a budding vesicle, (4) bead located near the ER, (5) bead in very tight interaction with the membrane. ER, endoplasmic reticulum; NGP, nanogold particle; TEM, transmission electron microscopy; Tf-LPA, Top-Fluor lysophosphatidic acid.

Finally, to confirm the results obtained with NGPs (Fig 5), we performed transmission electron microscopy (TEM) on SAG1 uptake using the conditions described above, using secondary antibodies coupled to 15-nm nanogold beads (secondary α-NGP; Fig 7). In good agreement with the results obtained above, three types of labelling were observed, with secondary α-NGP at the membrane (Fig 7A), accumulated (capped) at the basal pole of the parasite (Fig 7B), and endocytosed (Fig 7C and 7D). Consistent with the IFA quantification, membrane labelling was observed more regularly, followed by capped and then endocytosed SAG1. When internalised, secondary α-NGP were observed in translucent vesicles, as observed previously with NGP alone (Figs 5D3, 7C and 7D). As described for IFA, multiple areas on a single parasite could be labelled with the antibody, as illustrated in Fig 7E. Here, we can observe secondary α-NGP bound to the plasma membrane (panel 2), in very close contact with the plasma membrane, with a potential membrane invagination below (panel 5), endocytosed near the plasma membrane (panel 1), near the ER (panel 4), and inside small vesicles connected to the plasma membrane (panel 3).

## Discussion

### Extracellular parasites are capable of internalising exogenous material

Many proteins involved in endocytic uptake in other eukaryotes have been implicated in essential roles for trafficking of proteins to the unique secretory organelles of the parasite, giving rise to the hypothesis that apicomplexans repurposed their limited repertoire of trafficking factors to adapt to a parasitic lifestyle [53]. However, recent reports convincingly demonstrated uptake of host material during the intracellular development of the parasite and suggested that the endocytic pathway merges with the secretory pathway of the parasite [23,25]. This endocytic process occurs rapidly, with endocytosed proteins eventually reaching the *T*. *gondii* VAC [36,37] or *Plasmodium* food vacuole [25], in which they are digested [24]. In fact, McGovern and colleagues highlight an important point in their study [23]: endocytosis was always present, but fast protein degradation prevented classical methods of detection to work without using inhibitors. Here, we demonstrate that extracellular parasites, like intracellular parasites, are well capable of endocytosis and that the parasite appears to recycle surface proteins, such as SAG1 or micronemal proteins. Previous reports suggested that extracellular parasites are also capable of endocytosis, although only a minority of parasites within a population appear to take up material [54,55]. In good agreement, we demonstrate here that uptake of exogenous material, including surface proteins, occurs at a relatively low rate without stimulation, while lipid dyes are efficiently taken up, indicating efficient membrane recycling. Our attempts to perform live-cell imaging to measure the rate of uptake were so far unsuccessful due to phototoxicity. All experiments had to be performed in the dark, before fixation and visualisation of uptake.

Similar to the situation in intracellular parasites [24], we found that endocytosed material (NGP, phospholipids, or SAG1) colocalises with established markers of the endomembrane system of the parasite, such as ER, Golgi, VAC/PLV, and rhoptries, with a clear accumulation in the VAC/PLV. This strongly suggests that the same system is employed in intra- and extracellular parasites and that it converges with the secretory system of the parasite. In ultrastructural analysis, we validated this analysis and also identified several invaginations, where NGPs appear to enter the parasite. These invaginations are morphologically distinct from the previously identified micropore of the parasite [55] and were seen at different locations along the surface of the parasite, suggesting a dynamic system, in which the point of endocytosis can fluctuate. It is also worth mentioning that we failed to detect clathrin-coated pits at the point of entry.

To date, our attempts to obtain mechanistic insights regarding the endocytic process have been unsuccessful. While established endocytosis inhibitors show the expected effects, i.e., marked decrease in uptake of material, analysis of different DN mutants, such as dynamin, clathrin, or Rab-GTPases, did not show significant effects on endocytosis. While this might suggest that the analysed factors are not involved in endocytosis, it is possible that the conditional mutants used do not have the right kinetics for down-regulation of the respective genes, because this might already cause parasite death within the host cell, before extracellular endocytosis can be analysed. Therefore, faster conditional regulation systems should be used in future studies to re-analyse these factors, such as the auxin-inducible degron system [56].

### A link between gliding motility, retrograde membrane flow, secretion, and endocytosis

Retrograde membrane flow is implicated in many different motility modes of eukaryotic cells, and recent evidence demonstrates important roles for membrane trafficking in the regulation of cell migration in a variety of contexts. Indeed, findings made more than two decades ago demonstrated that all motile cells demonstrate the capping of surface antigens and, although sometimes regarded as artificially induced [57], recent studies demonstrated different important roles of the endocytic-secretory cycle. For example, the endocytic-secretory cycle is required for the internalisation and recycling of adhesion receptors, such as integrins or syndecans [58]. Another critical role is the maintenance of a constant cell surface [59,60], and recent studies demonstrated that the secretory-endocytic cycle can generate force for cell motility in an adhesion-independent way [15,16]. Importantly, amoeboid cells demonstrate rapid migration accompanied by rearward membrane flow, which was caused by increased endocytosis and membrane trafficking from back to front. O’Neill and colleagues, 2018, demonstrated that perturbation of polarised trafficking inhibited migration, and the ratio of cell migration and membrane flow was closely correlated [16].

The situation in *T*. *gondii* appears similar, and our results suggest that in addition to the acto-myosin motor complex, a fountain-flow-like model operates to generate retrograde membrane flow [57]. Previous studies implicated the generation of a retrograde membrane flow in parasite motility [5,11,17,61]. Surprisingly, interference with the acto-myosin system of the parasite did not abrogate bead translocation in *T*. *gondii* [5] or *Plasmodium* sporozoites [11,17], leading to the conclusion that retrograde membrane flow and force production can be uncoupled and that retrograde membrane flow can occur independently of parasite actin.

Using pharmacological and genetic disruption of the secretory-endocytic cycle, we demonstrate here a link between endocytosis, secretion, generation of retrograde membrane flow, and ultimately the gliding motility of the parasite, strongly suggesting that the fountain-flow model applies to *T*. *gondii*.

It is still unclear how the latex beads interact with the surface of the parasite. In our experiments, the latex beads have not been pretreated, because no specific receptor-ligand interactions are known. Again, these results correlate well with Quadt and colleagues, 2018, in which no differences between naked and streptavidin-coated beads were observed when analysing pulling forces using a laser trap [17].

The impact of Endo-buffer and DrpB DN on bead binding (Fig 2G) suggests that this interaction is dependent on micronemal proteins/material present at the plasma membrane. However, the exact mechanisms involved in this interaction(s) have still to be elucidated.

### The fountain-flow model and motility systems in apicomplexan parasites

Just like other eukaryotes, apicomplexan parasites have to move in different environments that exert different strains that the parasites need to overcome. It is possible that under some conditions (for example, 2D surfaces) the parasites’ glideosome is the main mode of action to ensure gliding motility, while in 3D or constricted environments, other motility modes become more important. Recent studies on cell migration in other eukaryotes combined reverse genetics, biophysics, mathematical modelling, and biomimetics to describe several novel mechanisms for cell motility, from more traditional amoeboid systems that depend on actin-myosin generated force, to fountain-flow, which depends on secretion-endocytosis or osmotic engines that depend on water permeation within a constricted environment [15,16,58,62–65]. Here, we provide compelling evidence that apicomplexans can, in addition to the glideosome, employ at least one alternative motility system that depends on secretion and endocytosis, strongly supporting the fountain-flow model [15]. However, it would not be surprising if future studies would discover additional motility mechanisms for apicomplexans. In fact, using mathematical modelling, we previously suggested that a gelsolation model is, in principle, a possible mode of motility [9], but to date, experimental evidence supporting this mode of action is missing. Furthermore, we do not believe that the different motility modes are mutually exclusive within the same environment, which is also reflected by the fact that residual motility is observed upon disruption of *T*. *gondii*’s acto-myosin system, both in 2D and 3D environments [5]. Indeed, it is likely that fountain-flow and acto-myosin act highly synergistically during parasite motility and might very well depend on each other. Both motility models depend on the secretion of micronemes—in one case, to generate membrane flow, and in the other case, to deposit micronemal proteins that act as force transmitters for the glideosome.

**Fig 8:**
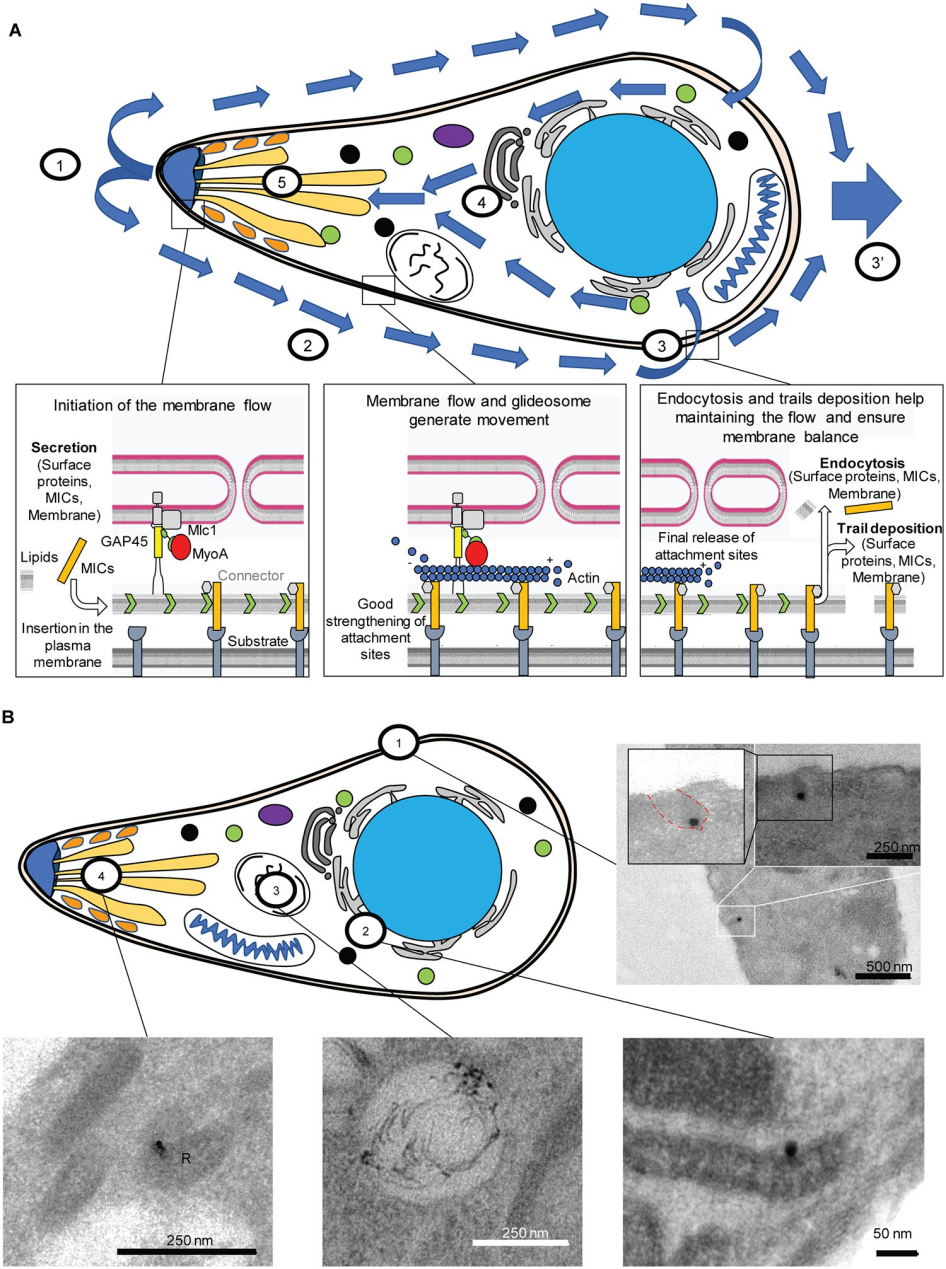
Fountain-flow model suggested for *T*. *gondii*. (A) General overview model of the fountain-flow applied to *T*. *gondii*. (1) Secretion from the secretory organelles at the apical end. (2) Retrograde flow allows bead translocation along the plasma membrane. (3, 3′) After translocation, membrane material can be either recycled (3) or left in a trail/cleaved (3′). (4) Endocytosed material is trafficked inside the parasite along the secretory system and accumulates in the VAC. (5) Incorporated lipids can be secreted in a complete endocytic-secretory cycle. (B) Summary of the different locations observed in TEM. (1) Entry from the plasma membrane via an invagination. (2) Perinuclear localisation, likely to be ER or Golgi. (3) Trafficking to the VAC. (4) Trafficking to the secretory organelles (rhoptry, R). ER, endoplasmic reticulum; TEM, transmission electron microscopy; VAC, vacuolar-like compartment.

Regarding the apicomplexan fountain-flow system, we propose the following mode of action, based on the presented data (Fig 8A): apical microneme secretion (1) initiates retrograde membrane flow (2), where excess membrane (and proteins) needs to be either recycled (2) or shed in surface trails (3′). The endocytosed vesicles converge with the parasites’ secretory system and are transported to different organelles, including the secretory organelles, closing the cycle (Fig 8B).

### Outlook and summary

While we demonstrate here an important role of secretion and endocytosis for the generation of retrograde membrane flow, which is required during gliding motility of apicomplexan parasites, we see this study as the first scratch on the surface and think the following questions need to be addressed in future studies: (1) we believe that apicomplexans—like other eukaryotes—can employ different modes of motility, depending on the environment in which they have to move. (2) At this point, we are not able to discriminate between the relative involvement of the acto-myosin system versus fountain-flow during motility, which will require future biophysical studies. (3) It is unclear which surface molecules, apart from SAG1, are recycled during motility. (4) The activation of endocytosis by LPA needs to be further analysed. (5) The trafficking factors involved in endocytosis need to be identified. It is likely that the parasite evolved specific structures for endocytic uptake, as suggested by the presence of the invagination seen in TEM analysis. A recent genome-wide screen [66] demonstrated the essentiality of hundreds of hypothetical genes. Many of them might well be involved in an essential uptake pathway, required for gliding motility and host cell invasion. Therefore, with the establishment of a reliable uptake assay, it is now possible to phenotypically screen for essential genes involved in this process, which will not only result in a fundamental understanding of this process but also in the identification of novel intervention strategies.

## Materials and methods

### Cloning DNA constructs

All primers used in this study are listed in S2 Table and were synthesised by Eurofins (Wolverhampton, United Kingdom).

### Culturing of parasites and host cells

Human foreskin fibroblasts (HFFs) were grown on TC-treated plastics and maintained in Dulbecco’s Modified Eagle Medium (DMEM) supplemented with 10% fetal bovine serum, 2 mM L-glutamine, and 25 mg/mL gentamycin. Parasites were cultured on HFFs and maintained at 37 °C and 5% CO_2_.

### *T*. *gondii* transfection and selection

To generate stable parasite lines, 1 × 10^7^ freshly lysed RH *Δhxgprt* or RH-DiCre Δ*ku80* parasites were transfected with 20 μg of DNA by AMAXA electroporation. Drug selection was carried out with either mycophenolic acid and xanthine, as described in [67], or with bleomycin.

### Generation of parasite lines

The following strains have been previously produced and published: CHC-HA, destabilisation domain (dd)-DrpB_DN_, dd-CHC_DN_, *act1* conditional knockout (cKO), *mlc1* cKO, *myoA* KO, dd-Rab18-myc, dd-Rab4-myc, and dd-RAB2-myc [5,31–33].

### VPS35-HA (TGGT1_242660), VPS53-HA (TGGT1_297230)

C-terminal 3 × HA epitope endogenous tagging of the *vps35* and *vps53* genes was carried out by the ligation-independent cloning (LIC) strategy, as previously described [68]. Briefly, 15 μg of each plasmid was linearised by EcoRV (LIC *vps35-HA*) or PstI (LIC *vps53-HA*) within the homologous region for efficient homologous recombination and was transfected into *Δku80* parasites. The resultant transfectants were selected for clonal lines expressing VPS35-HA or VPS53-HA in the presence of 25 μg/mL mycophenolic acid and 40 μg/mL xanthine and subsequently cloned by limiting dilution. Specific integration was confirmed by analytical PCR on genomic DNA using primers upstream the homology region inserted in the LIC vector and a reverse primer binding the LIC HA region (S2 Table).

### Internal tagging of AP2α (TGGT1_272600) by transient CRISPR-Cas9 expression

sgRNA plasmids were generated by PCR amplification of the guide RNA into a pU6-DHFR plasmid using Q5 mutagenesis kit and following manufacturer procedures (NEB, Ipswich, MA). RH Δ*ku80*Δ*hxgpt* parasites were transiently transfected using an AMAXA 4D Nucleofector (Lonza, Basel, Switzerland) with Cas9-YFP, each sgRNA plasmid, and reparation template PCR product. Briefly, a total of 10 μg of precipitated plasmid DNA and PCR product and approximately 9 × 10^5^ parasites were resuspended in 20 μL of Buffer P3 (P3 Primary Cell 4D Nucleofector X kit S [32 RCT], Lonza, Basel, Switzerland). Parasites were transfected in a multi-well format using programme FI-158, transferred to an HFF monolayer, and fixed at 24 and 48 hours post transfection. Parasites were considered transfected if expressing GFP-Cas9 in the nucleus. Checking of the integration was made as described above.

### Inducing conditional knockdown lines and protein expression

dd-DrpB_DN_, dd-CHC_DN_, dd-DrpB_DN_, dd-Rab18-myc, dd-Rab4-myc, and dd-RAB2-myc parasites (S1 Table) were grown until the vacuoles were ready to lyse. Shield was added 6 hours prior to the parasites being used for experiments. *act1* cKO and *mlc1* cKO were induced as previously described [5].

### Phenotypic characterisations

#### Trail deposition assay

Gliding assays were performed as described before [5]. Briefly, freshly lysed parasites were allowed to glide on FBS-coated glass slides for 30 minutes before they were fixed with 4% paraformaldehyde (PFA) and stained with α-SAG1 under nonpermeabilising conditions. The mean values of three independent experiments ± SD were determined. Where drugs were used, parasites were pre-incubated for 10 minutes in the respective concentration before the start of the assay: 0.5 μM CD (Sigma, St. Louis, MO), 10 μM phenyl arsine oxide (Sigma, St. Louis, MO), or 50 μM trifluoperazine dihydrochloride (TFDC) (Sigma, St. Louis, MO). The same concentrations were used in the different assays.

#### Secretion assays

Microneme secretion was analysed by monitoring the release of MIC2 into the culture medium, as described previously (Huynh and colleagues, 2006). The effect of Tf-LPA on both constitutive and induced secretion was evaluated.

#### Two-dimensional motility assay

Time-lapse video microscopy was used to analyse the kinetics over a 2D surface, similarly as previously described [6]. Briefly, Ibidi μ-dish^35mm-high^ was coated in 100% FBS for 2 hours at room temperature. Freshly egressed parasites were added to the dish. Time-lapse videos were taken with a 40× objective at 1 frame per second using a DeltaVision Core microscope. Analysis was made using ImageJ wrMTrckr tracking plugin. For analysis, 20 parasites were tracked during both helical and circular trails, with the corresponding distance travelled and average and maximum speeds determined. Mean values of three independent experiments ± SD were determined.

#### Invasion assay

For the assay, 5 × 10^4^ freshly lysed parasites were allowed to invade a confluent layer of HFFs for 1 hour after 30 minutes of treatment with or without Tf-LPA. Subsequently, five washing steps were performed for removal of extracellular parasites. Cells were then incubated for a further 24 hours before fixation with 4% PFA. Afterwards, parasites were stained with the α-IMC1 antibody [9]. The number of vacuoles in 15 fields of view was counted. Mean values of three independent experiments ± SD were determined.

#### Capping assays

Capping assays were performed as previously described [5]. Briefly, Ibidi live cell dishes (29 mm) were coated with 0.1% poly-L-Lysine for 30 minutes and washed with MilliQ water. Fluorescent latex beads (FluoSpheres, 0.04 μm, Invitrogen, Carlsbad, CA) were diluted at 5 μL in 400 μL of a mixture of Hanks Balanced Salt Solution (HBSS) and HEPES (25 mM; described hereafter as H-H buffer). After a short spin (10 seconds, 6,000*g*), the supernatant was recovered and left on ice for 30 minutes before use. Parasites were harvested, pelleted, and resuspended in cold H-H buffer to achieve 10^7^ parasites/mL. Parasites were then transferred to poly-L-Lysine coated dishes and left on ice for 10 minutes. A total of 5 μL of diluted beads was added to 250 μL of H-H buffer and added to the parasites. Immediately, the dish was incubated at 37 °C for 30 minutes. The experiment was stopped by the addition of 2 mL of 4% PFA and incubated at 4 °C for 10 minutes. The PFA was washed gently and parasite nuclei stained with Hoechst 0.01%. For time course analysis, parasites were fixed at different time points after the addition of the beads. For the drug and buffer assays, parasites were incubated for 10 minutes in the buffer of interest before their incubation on the coated dish. In these cases, all other experimental components were also diluted using the same buffer. For each experiment (*n*), an average of 1,000 parasites were analysed. Total numbers of parasites, number of parasites without beads, with beads bound, and with beads capped were quantified. Mean values of three independent experiments ± SD were determined.

#### Live capping assays

Capping assays were adapted for live microscopy. Parasites were prepared as described above. After the addition of the diluted beads (5 μL of beads in 250 μL of H-H buffer) to the parasites, the dish was incubated for 10 minutes on ice. After incubation, the media was exchanged for 500 μL of ice-cold H-H buffer without beads. The dish was then directly transferred to the microscope. Time-lapse videos were taken with a 60× objective at 1 frame per second using a DeltaVision Core microscope. Analysis was made using ImageJ.

#### Tf-LPA, Tf-LPC, Bodipy, and NGP uptake

Tf-LPA (Avanti Polar Lipids, Alabaster, AL), Tf-LPC (Avanti Polar Lipids, Alabaster, AL), bodipy (BODIPY 493/503, Thermo Fisher, Waltham, MA), and NGPs (Gold Nanoparticles 10 nM Cy5.5 labelled, Nanocs, New York, NY) uptake assays were derived from the capping assays. Briefly, Ibidi live cell dishes (29 mm) were coated with 0.1% poly-L-Lysine for 30 minutes and washed with MilliQ water. NGPs were diluted at 8 μL in 400 μL H-H buffer and left on ice for 30 minutes before use. Tf-LPA, Tf-LPC, and Bodipy were diluted to a concentration of 4 μM in H-H buffer. Parasites were harvested, pelleted, and resuspended in cold H-H Buffer to achieve 10^7^ parasites/mL. Parasites were then transferred to poly-L-Lysine–coated dishes and left on ice for 20 minutes. A total of 250 μL of H-H buffer (±4 μM Tf-LPA, Tf-LPC, or Bodipy), with or without 8 μL of diluted beads, was added to the parasites. Immediately, the dish was incubated at 37 °C for 30 minutes. The experiment was stopped by addition of 2 mL of 4% PFA and incubated at 4 °C for 10 minutes. The PFA was washed gently and parasite nuclei stained with Hoechst 0.01%. For time course analysis, parasites were fixed at different time points (0, 1, 5, 10, 15, 20, and 30 minutes) after the addition of the beads. For the drug and buffer assays, parasites were incubated for 10 minutes in the buffer of interest before their incubation on the coated dish. Mean values of three independent experiments ± SD were determined.

#### αSAG1/MIC6/MIC8 uptake

RH parasites were incubated with α-SAG1 on Ibidi live cell dishes (29 mm) coated with neat FBS for 1 hour at 4 °C. After that, three washes with ice-cold PBS were performed, and the secondary antibodies (Alexa-350) were incubated with the parasites for 1 hour at 4 °C. After washing again, Tf-LPA was or was not added as described above. After 30 minutes of incubation at 37 °C/4 °C, parasites were fixed and uptake evaluated. Mean values of three independent experiments ± SD were determined. For MICs, the same experiment was done with a stimulation with 2 μM of calcium ionophore for 5 minutes at 37 °C prior the incubation with the primary antibodies. For αSAG1 uptake TEM, secondary antibodies were coupled with 15-nm gold beads instead of Alexa dye.

#### Tf-LPA secretion evaluation

RH parasites were treated for 30 minutes with Tf-LPA (2 μM final) with or without particles, as described above. After 30 minutes, the media was exchanged twice and parasites washed thoroughly to collect and transfer them to a new dish with either H-H buffer (MM), DMEM supplemented with 10% fetal bovine serum, 2 mM L-glutamine and 25 mg/mL gentamycin (CM), or coverslips with host cell in CM (Host cell). After 30 minutes of incubation, parasites were fixed and the presence of Tf-LPA and NGP was evaluated. For parasites on host cells, a dual SAG1 (without permeabilisation) and GAP45 (with permeabilisation) was carried out to differentiate invaded from extracellular parasites. Mean values of three independent experiments ± SD were determined and compared to a control fixed at the end of the first 30 minutes of incubation with Tf-LPA and NGP (*t* = 0).

#### Evacuoles formation

Assay performed as previously described [45]. Briefly, after incubating parasites with Tf-LPA for 30 minutes as described above and washing with ice-cold PBS, parasites were treated with 1 μM CytD for 10 minutes at room temperature. They were then transferred to an HFF monolayer for 10 minutes at 37 °C in the presence of 1 μM CytD; the monolayers were rinsed in PBS prior to PFA fixation. IFA using ROP1 antibodies was done to detect the formation of evacuoles.

#### Plaque assay

Parasites were treated for 30 minutes with or without Tf-LPA (2 μM Final). A total of 1 × 10^3^ parasites were inoculated on a confluent layer of HFFs and incubated for 5 days, after which the HFFs were washed once with PBS and fixed with ice-cold MeOH for 20 minutes. HFFs were stained with Giemsa, with plaque area measured using Fiji software. Mean values of three independent experiments ± SD were determined.

#### Immunofluorescence analysis

Immunofluorescence analysis was carried out as previously described [9]. Briefly, parasites were fixed in 4% paraformaldehyde for 10 minutes at 4 °C. Afterwards, coverslips were blocked and permeabilised in 2% BSA and 0.2% Triton X–100 in PBS for 20 minutes. The staining was performed using the indicated combinations of primary antibodies for 1 hour, followed by the incubation with secondary AlexaFluor 350, AlexaFluor 488, AlexaFluor 594, or AlexaFluor 633 conjugated antibodies (1:3,000, Invitrogen, Carlsbad, CA) for another 45 minutes, respectively. For quantification, the mean values of three independent experiments ± SD were determined. For time course IFA, parasites were fixed at different time points (5, 10, 15, 20, and 30 minutes) after the addition of the beads and imaged as described above.

#### SIM imaging

Super-resolution structure illumination microscopy (SR-SIM) was carried out using an ELYRA PS.1 microscope (Zeiss, Oberkochen, Germany). Images were acquired using a Plan Apochromat 63×, 1.4 NA oil immersion lens, recorded with a CoolSNAP HQ camera (Photometrics, Tucson, AZ), and analysed using ZEN Black software (Zeiss, Oberkochen, Germany) and ImageJ software.

#### TEM of NGP

Extracellular parasites (±Tf-LPA/NGP, see NGP uptake above) were fixed with 2.5% glutaraldehyde in 0.1 M phosphate buffer, pH 7.4, after the indicated incubation. Samples were processed for routine electron microscopy as described previously [69] and examined in a JEOL 1200EX electron microscope.

#### CLEM

Uptake assays were carried out in gridded glass bottom petri dishes (MatTek, Ashland, MA). Parasites presenting clear Tf-LPA and NGP uptake were imaged with SR-SIM in an ELYRA PS.1 microscope (Zeiss, Oberkochen, Germany), and the material was fixed in 2.5% glutaraldehyde and 4% paraformaldehyde in 0.1 M phosphate buffer and processed for TEM, as described previously [69]. Thin sections of the same areas imaged in three-dimensional structure illumination microscopy (3D-SIM) were imaged in a Tecnai T20 transmission electron microscope (FEI, the Netherlands).

#### TEM of SAG1 labelling with secondary antibody conjugated to 15-nm gold beads

Extracellular parasites (±LPA/αSAG1, see αSAG1 uptake above) were fixed with 2.5% (v/v) glutaraldehyde in 0.1 M phosphate buffer, pH 7.4, after the indicated incubation. The parasites were washed three times at room temperature with PBS (137 mM NaCl_2_, 2.7 mM KCl, 10 mM Na_2_HPO_4_, 1.8 mM KH_2_PO_4_, pH 7.4) and postfixed with 1% (w/v) osmium tetroxide for 1 hour. Subsequent to washing with PBS and water, the samples were stained en bloc with 1% (w/v) uranyl acetate in 20% (v/v) acetone for 30 minutes. Samples were dehydrated in a series of graded acetone and embedded in Epon 812 resin. Ultrathin sections (thickness, 60 nm) were cut using a diamond knife on a Reichert Ultracut-E ultramicrotome. Sections were mounted on collodium-coated copper grids, post-stained with lead citrate (80 mM, pH 13), and examined with an EM 912 transmission electron microscope (Zeiss, Oberkochen, Germany) equipped with an integrated OMEGA energy filter operated in the zero-loss mode at 80 kV. Images were acquired using a 2k × 2k slow-scan CCD camera (Tröndle Restlichtverstärkersysteme, Moorenweis, Germany).

## Supporting information

S1 Fig Uptake of FM-dyes, Cell-Mask, and phospholipids. (A) Representative pictures of FM-dye 64FX uptake upon a temperature shift from 4 °C to 37 °C. The experiment was done on RH parasites expressing cytosolic GFP. (B) Representative pictures of Cell-Mask uptake upon a temperature shift from 4 °C to 37 °C. The experiment was done on RH parasites expressing cytosolic GFP. (C) Uptake of phospholipids: extended version of Fig 2A. Tf-LPA, Tf-LPC, and Bodipy were analysed at 37 °C and 4 °C. Incubation at 37 °C demonstrates the uptake of all tested molecules. Mean values of three independent assays are shown ± SEM. ***p < 0.001 in a two-tailed Student *t* test. (Right panels) Examples of images obtained for the quantification, as shown in the graph. Scale bar, 1 μm. For each bar graph, the corresponding data can be found in S1 Data. GFP, green fluorescent protein; Tf-LPA, Top-Fluor lysophosphatidic acid; Tf-LPC, Top-Fluor lysophosphatidyl choline.
(TIF)

S2 Fig Tf-LPA does not impact *T*. *gondii* morphology and fitness. (A) TEM comparison of RH- and Tf-LPA–treated parasites. No difference was observed between Tf-LPA− and Tf-LPA+ parasites. (B) Constitutive secretion assay. Secretion of MIC2 was tested for both RH and RH Tf-LPA parasites in CM after 30 minutes. Treatment with Tf-LPA does not impact secretion or processing of MIC2. (C) Induced secretion assay. Secretion of MIC2 was tested for both RH, RH + Tf-LPA, and RH + calcium ionophore A23187 in MM for 5 minutes. Treatment with Tf-LPA does not impact secretion of MIC2 in opposition to calcium ionophore, which stimulates the secretion, as expected. (D) Invasion rate of parasites treated with or without Tf-LPA. Tf-LPA did not impact parasite invasion. (E) Treatment with Tf-LPA did not impact parasite growth, as determined by plaque assay. Incubation of the parasite with Tf-LPA did not impact the number or the size of the plaques, illustrating that the molecule is not toxic. For each bar graph, the corresponding data can be found in S1 Data. TEM, transmission electron microscopy; Tf-LPA, Top-Fluor lysophosphatidic acid.
(TIF)

S3 Fig Supplementary IFA localisations and control. (A) Extended colocalisation analysis, as shown in Fig 4. Here, examples of ‘no-colocalisation’ are shown. No colocalisation was observed with Rab2, MIC2, or GRA-1. Scale bar, 1 μm. (B) Comparison of the tested IFA conditions between RH and RH + Tf-LPA. Scale bar, 1 μm. No signal alteration was observed by Tf-LPA addition. (C) IFA of an invaded parasite using anti-ROP1 antibodies. Tf-LPA, Top-Fluor lysophosphatidic acid.
(TIF)

S4 Fig Tf-LPA can be secreted. (A) Representative images of parasites transferred into MM or CM. *t* = 0 represents the parasite after the initial 30-minute uptake. At *t* = 30 minutes, MM led to a phenotype similar to *t* = 0. In the case of CM, the number of positive vesicles was clearly reduced (CM1), and some parasites showed a different labelling (CM2). Scale bar, 1 μm. (B) Illustration of parasites transferred onto host cells. SAG1 staining (prior to permeabilisation) was used to differentiate intra- from extracellular parasites. Two intracellular parasites are shown: (1) Parasite invaded with internal Tf-LPA vesicles, (2) parasite invaded without internal Tf-LPA vesicles. Scale bar, 1 μm. CM, complete media; MM, minimal media; Tf-LPA, Top-Fluor lysophosphatidic acid.
(TIF)

S5 Fig Uptake material is observed below the plasma membrane. Illustration of the uptake of NGP in RH without Tf-LPA stimulation. Parasites were imaged by 3D-SIM microscopy. Scale bar, 1 μm. Green, αGAP45; red, NGP; blue, nucleus. Z-slice of a parasite showing NGP uptake and its respective Ortho-view clearly showing the accumulation below IMC (αGAP45) and therefore the uptake in NGP in unstimulated parasites. Scale bar, 1 μm. (B) Illustration of the simultaneous presence of different types of the labelling (membrane, capped, uptake) observed during αSAG1 uptake assay. Scale bar, 1 μm. (C) Z-slice of a parasite showing αSAG1 (blue) uptake and its respective Ortho-view clearly showing the accumulation below IMC (αGAP45, red). Green Tf-LPA. Scale bar, 1 μm. (D) Z-slice of a parasite showing αMIC6 (blue) uptake and its respective Ortho-view clearly showing the accumulation below the plasma membrane (αSAG1, red). Green Tf-LPA. Scale bar, 1 μm. (E) Z-slice of a parasite showing αMIC8 (blue) uptake and its respective Ortho-view clearly showing the accumulation below the plasma membrane (αSAG1, red). Green Tf-LPA. Scale bar, 1 μm. (F) Z-slice of a parasites showing ConA (blue) uptake and its respective Ortho-view clearly showing the accumulation below IMC (αGAP45, red). ConA, Concanavalin A; IMC, inner membrane complex; NGP, nanogold particle; Tf-LPA, Top-Fluor lysophosphatidic acid; 3D-SIM, three-dimensional structure illumination microscopy.
(TIF)

S1 Table Markers used to characterise the nature of the vesicles labelled with Tf-LPA and/or NGP. We used different antibodies or parasite strains to localise different organelles within the cell. Parasite strains with overexpressed marker are highlighted in blue (second copy tagged) and endogenously tagged proteins in orange. Parasite strains reported for the first time are indicated with an asterisk (*). NGP, nanogold particle; Tf-LPA, Top-Fluor lysophosphatidic acid.
(XLSX)

S2 Table List of primers. List of primers used in the cloning and confirmation of integration for the strains VPS35-HA (Retromer complex), VPS53-HA (GARP complex), and Adaptor protein AP2-HA.
(XLSX)

S1 Video Capping in wild-type parasites. (AVI)

S2 Video Gliding of parasites after translocation of beads. (AVI)

S3 Video Loss of capped beads in gliding parasites. (AVI)

S4 Video Accumulation of Tf-LPA in capped parasites. Tf-LPA, Top-Fluor lysophosphatidic acid.
(WMV)

S5 Video Localisation of NGP within vesicles. NGP, nanogold particle.
(AVI)

S1 Data Raw data were regrouped in a single excel file. Each sheet is named after its corresponding figure.
(XLSX)

## Abbreviations

3D-SIM: three-dimensional structure illumination microscopy
AFU: arbitrary fluorescent unit
CD: Cytochalasin D
CDE: clathrin-dependent endocytosis
CHC: clathrin heavy chain
CIE: clathrin-independent endocytosis
cKO: conditional knockout
CLEM: correlative light and electron microscopy
CM: complete media
ConA: Concanavalin A
CPL: cathepsin L
dd: destabilisation domain
DMEM: Dulbecco’s Modified Eagle Medium
DN: dominant negative
DrpB: dynamin-related protein B
ELC: endosome-like compartment
ER: endoplasmic reticulum
ESCRT: endosomal sorting complexes required for transport
HBSS: Hanks Balanced Salt Solution
HFF: human foreskin fibroblast
IMC: inner membrane complex
LIC: ligation-independent cloning
MIC: microneme protein
MM: minimal media
NGP: nanogold particle
P.C.: phase contrast
PFA: paraformaldehyde
PLV: plantlike vacuole
SAG1: surface antigen 1
SR-SIM: super-resolution structure illumination microscopy
TEM: transmission electron microscopy
TFDC: trifluoperazine dihydrochloride
Tf-LPA: Top-Fluor lysophosphatidic acid
Tf-LPC: Top-Fluor lysophosphatidyl choline
TGN: *Trans*-Golgi network
VAC: vacuolar-like compartment
VPS: vacuolar protein sorting-associated protein
