# Supplementary figures and images for: An endocytic-secretory cycle participates in Toxoplasma gondii in motility
Source: PLoS Biol. 2019 Jun 24;17(6):e3000060. doi: 10.1371/journal.pbio.3000060 (PMC6611640; doi:10.1371/journal.pbio.3000060)

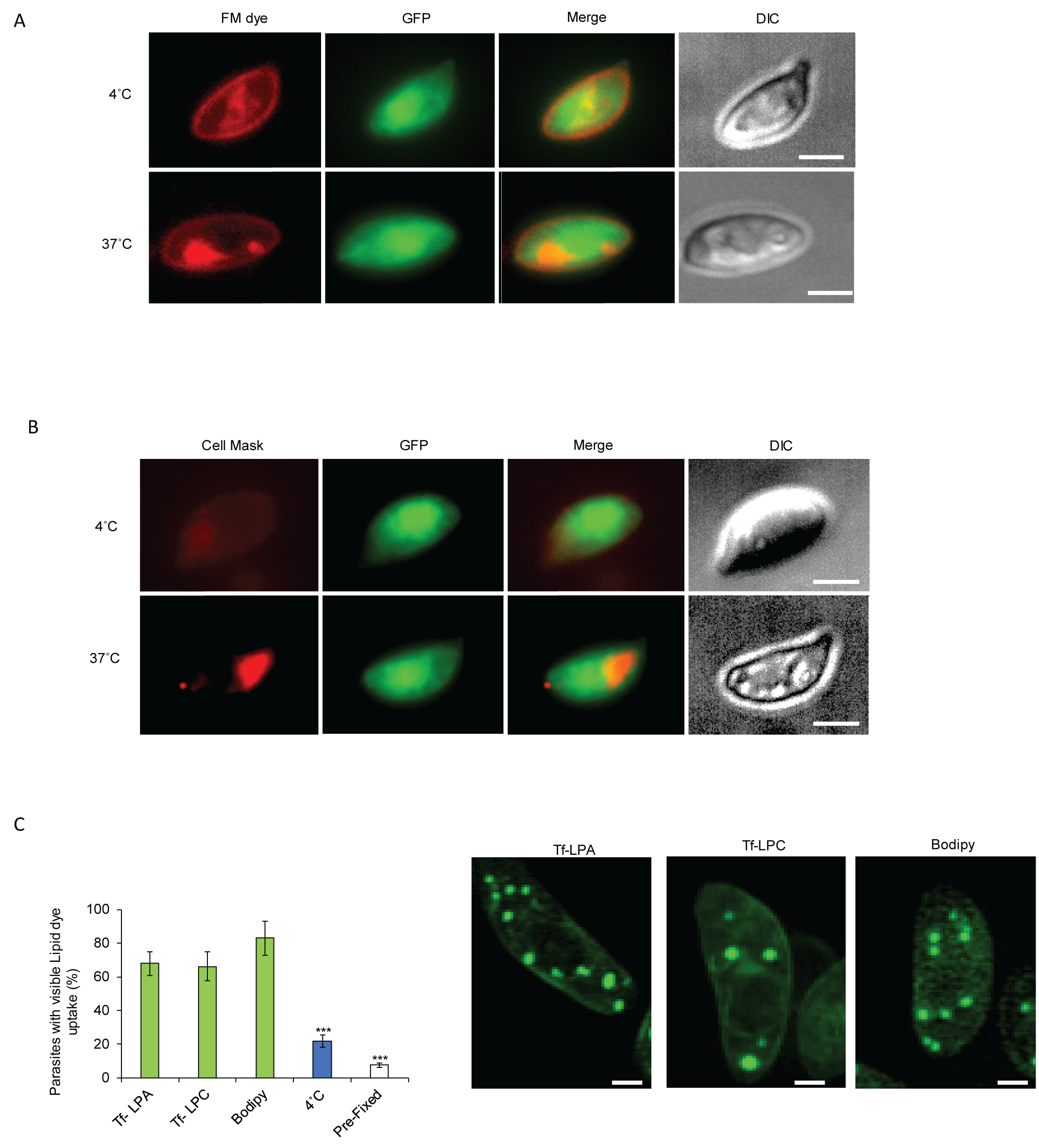

Supplement: S1 Fig — (A) Representative pictures of FM-dye 64FX uptake upon a temperature shift from 4 °C to 37 °C. The experiment was done on RH parasites expressing cytosolic GFP. (B) Representative pictures of Cell-Mask uptake upon a temperature shift from 4 °C to 37 °C. The experiment was done on RH parasites expressing cytosolic GFP. (C) Uptake of phospholipids: extended version of Fig 2A. Tf-LPA, Tf-LPC, and Bodipy were analysed at 37 °C and 4 °C. Incubation at 37 °C demonstrates the uptake of all tested molecules. Mean values of three independent assays are shown ± SEM. ***p < 0.001 in a two-tailed Student t test. (Right panels) Examples of images obtained for the quantification, as shown in the graph. Scale bar, 1 μm. For each bar graph, the corresponding data can be found in S1 Data. GFP, green fluorescent protein; Tf-LPA, Top-Fluor lysophosphatidic acid; Tf-LPC, Top-Fluor lysophosphatidyl choline. (TIF) [file pbio.3000060.s001.tif]

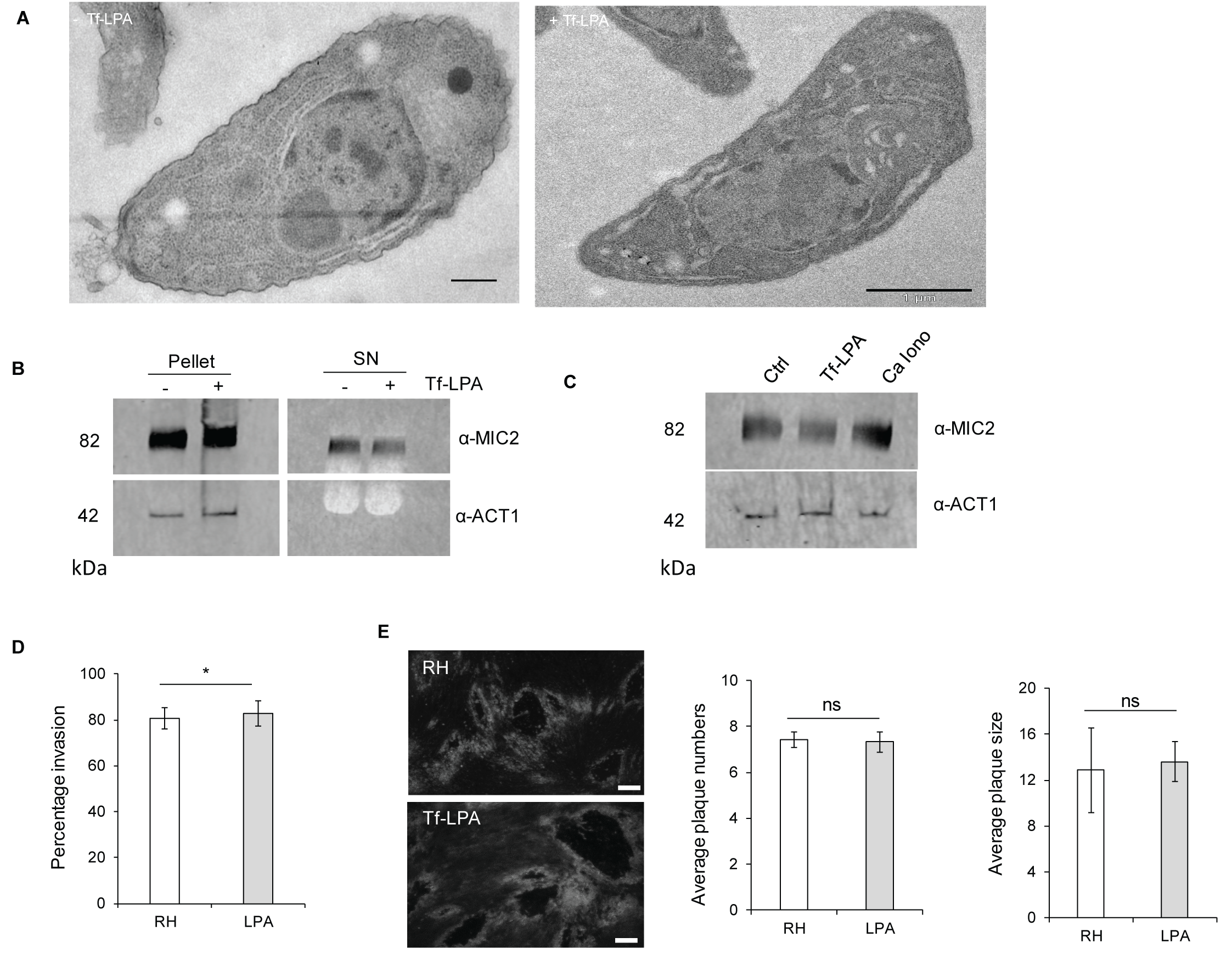

Supplement: S2 Fig — (A) TEM comparison of RH- and Tf-LPA–treated parasites. No difference was observed between Tf-LPA− and Tf-LPA+ parasites. (B) Constitutive secretion assay. Secretion of MIC2 was tested for both RH and RH Tf-LPA parasites in CM after 30 minutes. Treatment with Tf-LPA does not impact secretion or processing of MIC2. (C) Induced secretion assay. Secretion of MIC2 was tested for both RH, RH + Tf-LPA, and RH + calcium ionophore A23187 in MM for 5 minutes. Treatment with Tf-LPA does not impact secretion of MIC2 in opposition to calcium ionophore, which stimulates the secretion, as expected. (D) Invasion rate of parasites treated with or without Tf-LPA. Tf-LPA did not impact parasite invasion. (E) Treatment with Tf-LPA did not impact parasite growth, as determined by plaque assay. Incubation of the parasite with Tf-LPA did not impact the number or the size of the plaques, illustrating that the molecule is not toxic. For each bar graph, the corresponding data can be found in S1 Data. TEM, transmission electron microscopy; Tf-LPA, Top-Fluor lysophosphatidic acid. (TIF) [file pbio.3000060.s002.tif]

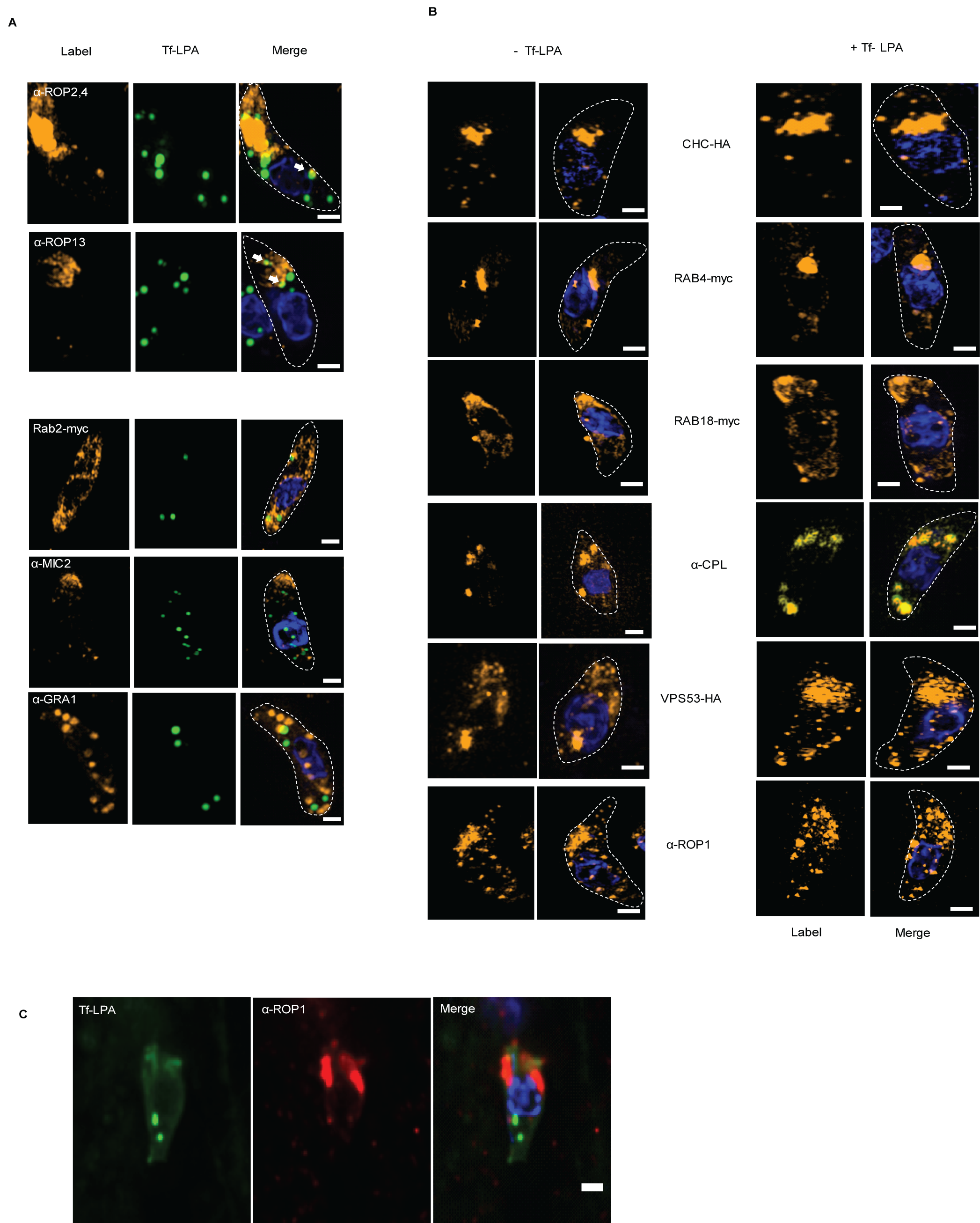

Supplement: S3 Fig — (A) Extended colocalisation analysis, as shown in Fig 4. Here, examples of ‘no-colocalisation’ are shown. No colocalisation was observed with Rab2, MIC2, or GRA-1. Scale bar, 1 μm. (B) Comparison of the tested IFA conditions between RH and RH + Tf-LPA. Scale bar, 1 μm. No signal alteration was observed by Tf-LPA addition. (C) IFA of an invaded parasite using anti-ROP1 antibodies. Tf-LPA, Top-Fluor lysophosphatidic acid. (TIF) [file pbio.3000060.s003.tif]

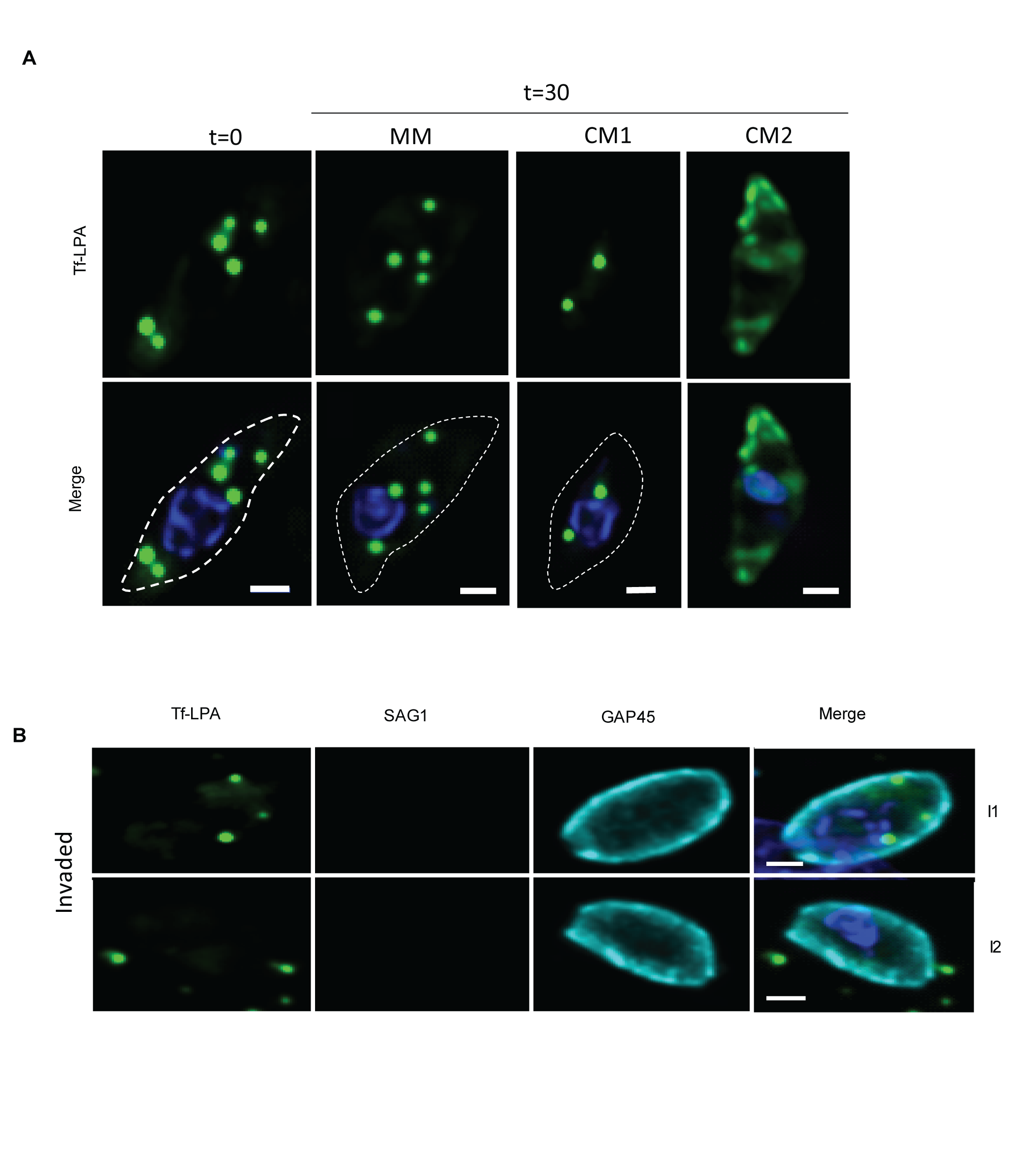

Supplement: S4 Fig — (A) Representative images of parasites transferred into MM or CM. t = 0 represents the parasite after the initial 30-minute uptake. At t = 30 minutes, MM led to a phenotype similar to t = 0. In the case of CM, the number of positive vesicles was clearly reduced (CM1), and some parasites showed a different labelling (CM2). Scale bar, 1 μm. (B) Illustration of parasites transferred onto host cells. SAG1 staining (prior to permeabilisation) was used to differentiate intra- from extracellular parasites. Two intracellular parasites are shown: (1) Parasite invaded with internal Tf-LPA vesicles, (2) parasite invaded without internal Tf-LPA vesicles. Scale bar, 1 μm. CM, complete media; MM, minimal media; Tf-LPA, Top-Fluor lysophosphatidic acid. (TIF) [file pbio.3000060.s004.tif]

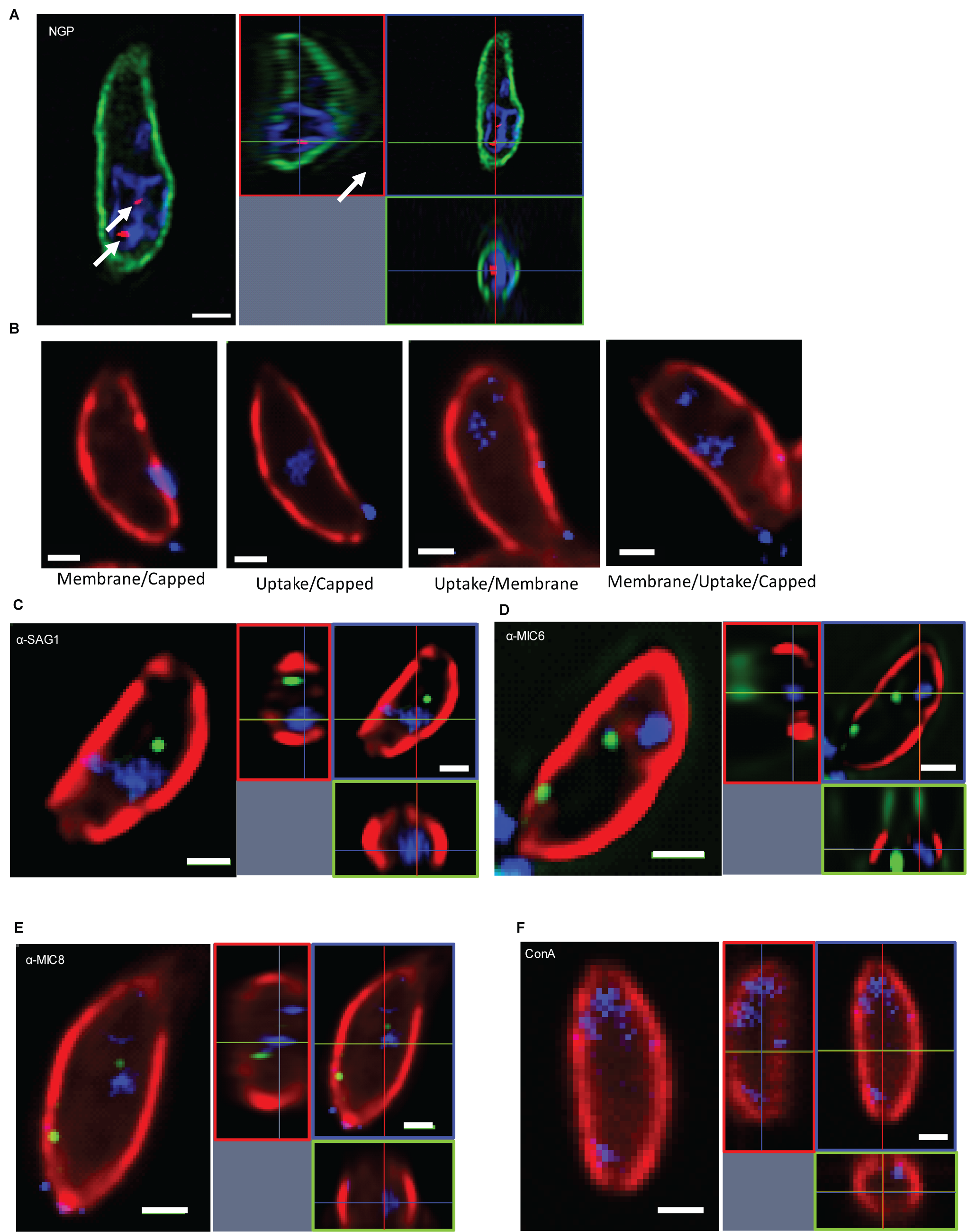

Supplement: S5 Fig — Illustration of the uptake of NGP in RH without Tf-LPA stimulation. Parasites were imaged by 3D-SIM microscopy. Scale bar, 1 μm. Green, αGAP45; red, NGP; blue, nucleus. Z-slice of a parasite showing NGP uptake and its respective Ortho-view clearly showing the accumulation below IMC (αGAP45) and therefore the uptake in NGP in unstimulated parasites. Scale bar, 1 μm. (B) Illustration of the simultaneous presence of different types of the labelling (membrane, capped, uptake) observed during αSAG1 uptake assay. Scale bar, 1 μm. (C) Z-slice of a parasite showing αSAG1 (blue) uptake and its respective Ortho-view clearly showing the accumulation below IMC (αGAP45, red). Green Tf-LPA. Scale bar, 1 μm. (D) Z-slice of a parasite showing αMIC6 (blue) uptake and its respective Ortho-view clearly showing the accumulation below the plasma membrane (αSAG1, red). Green Tf-LPA. Scale bar, 1 μm. (E) Z-slice of a parasite showing αMIC8 (blue) uptake and its respective Ortho-view clearly showing the accumulation below the plasma membrane (αSAG1, red). Green Tf-LPA. Scale bar, 1 μm. (F) Z-slice of a parasites showing ConA (blue) uptake and its respective Ortho-view clearly showing the accumulation below IMC (αGAP45, red). ConA, Concanavalin A; IMC, inner membrane complex; NGP, nanogold particle; Tf-LPA, Top-Fluor lysophosphatidic acid; 3D-SIM, three-dimensional structure illumination microscopy. (TIF) [file pbio.3000060.s005.tif]
